# Zinc Finger Independent Genome-Wide Binding of Sp2 Potentiates Recruitment of Histone-Fold Protein Nf-y Distinguishing It from Sp1 and Sp3

**DOI:** 10.1371/journal.pgen.1005102

**Published:** 2015-03-20

**Authors:** Sara Völkel, Bastian Stielow, Florian Finkernagel, Thorsten Stiewe, Andrea Nist, Guntram Suske

**Affiliations:** 1 Institute of Molecular Biology and Tumor Research (IMT), Philipps-University of Marburg, Marburg, Germany; 2 Genomics Core Facility, Zentrum für Tumor- und Immunbiologie (ZTI), Philipps-University of Marburg, Marburg, Germany; The University of North Carolina at Chapel Hill, United States of America

## Abstract

Transcription factors are grouped into families based on sequence similarity within functional domains, particularly DNA-binding domains. The Specificity proteins Sp1, Sp2 and Sp3 are paradigmatic of closely related transcription factors. They share amino-terminal glutamine-rich regions and a conserved carboxy-terminal zinc finger domain that can bind to GC rich motifs *in vitro*. All three Sp proteins are ubiquitously expressed; yet they carry out unique functions *in vivo* raising the question of how specificity is achieved. Crucially, it is unknown whether they bind to distinct genomic sites and, if so, how binding site selection is accomplished. In this study, we have examined the genomic binding patterns of Sp1, Sp2 and Sp3 in mouse embryonic fibroblasts by ChIP-seq. Sp1 and Sp3 essentially occupy the same promoters and localize to GC boxes. The genomic binding pattern of Sp2 is different; Sp2 primarily localizes at CCAAT motifs. Consistently, re-expression of Sp2 and Sp3 mutants in corresponding knockout MEFs revealed strikingly different modes of genomic binding site selection. Most significantly, while the zinc fingers dictate genomic binding of Sp3, they are completely dispensable for binding of Sp2. Instead, the glutamine-rich amino-terminal region is sufficient for recruitment of Sp2 to its target promoters *in vivo*. We have identified the trimeric histone-fold CCAAT box binding transcription factor Nf-y as the major partner for Sp2-chromatin interaction. Nf-y is critical for recruitment of Sp2 to co-occupied regulatory elements. Equally, Sp2 potentiates binding of Nf-y to shared sites indicating the existence of an extensive Sp2-Nf-y interaction network. Our results unveil strikingly different recruitment mechanisms of Sp1/Sp2/Sp3 transcription factor members uncovering an unexpected layer of complexity in their binding to chromatin *in vivo*.

## Author Summary

A major question in eukaryotic gene regulation is how transcription factors with similar structural features elicit specific biological responses. We used the three transcription factors Sp1, Sp2 and Sp3 as a paradigm for investigating this question. All three proteins are ubiquitously expressed, and they share glutamine-rich domains as well as a conserved *bona fide* zinc finger DNA binding domain. Yet, each of the three proteins carries out unique functions *in vivo*, and each is absolutely essential for mouse development. By genome-wide binding analysis, we found that Sp1 and Sp3 on the one hand, and Sp2 on the other hand engage completely different protein domains for their genomic binding site selection. Most strikingly, the zinc finger domain of Sp2 is dispensable for recruitment to its target sites *in vivo*. Moreover, we provide strong evidence that the histone-fold protein Nf-y is necessary for recruitment of Sp2. Conversely, Sp2 potentiates Nf-y binding showing that binding of Sp2 and Nf-y to shared sites is mutually dependent. Our findings uncover an unexpected mechanistic diversity in promoter recognition by seemingly similar transcription factors. This work has broader implications for our understanding of how members of other multi-protein transcription factor families could achieve specificity.

## Introduction

Eukaryotic transcription factors are grouped into families based on their common structural features. Prototypical zinc finger-containing transcription factors are the evolutionary conserved Specificity proteins/Krüppel-like factors (Sps/Klfs) (reviewed in [1,2,3,4]) that share three consecutive C2H2-type zinc fingers in their C-terminal moiety. Mammals have nine different Sp factors (Sp1 to Sp9), which can be grouped into two subclasses based on structural features outside of the zinc finger domain [5]. The Sp1 to Sp4 subclass is characterized by glutamine-rich domains that have been shown to act as transactivation domains in Sp1, Sp3 and Sp4. Sp1, Sp2 and Sp3 are ubiquitously expressed whereas expression of Sp4 is largely restricted to neuronal cells [1,6].

Despite their structural similarities and broad co-expression there seems to be little functional overlap between Sp1, Sp2 and Sp3 [7,8,9]. Briefly, Sp1*null* as well as Sp2*null* embryos are severely growth-retarded and die before embryonic day 10 [7,9]. Conditional inactivation of Sp2 in neuronal stem cells and neuronal progenitor cells resulted in impaired proliferation and disrupted neurogenesis in embryonic and postnatal brain [10]. In homozygous Sp2 transgenic mice terminally differentiated keratinocytes are depleted and the animals die within two weeks after birth again underlining the physiological importance of Sp2 [11]. Moreover, a genome-wide screen for cell division genes identified *Sp2* as a gene essential for proper mitosis in HeLa cells [12]. Finally, Sp3*null* mice develop until birth but are not viable due to manifold defects including impaired lung, cardiac, bone and red blood cell development [8,13,14,15].

At the molecular level, the functional properties of Sp1 and Sp3 are well characterized. Particularly, numerous publications reported binding of Sp1 and Sp3 to the GC box (GGGGCGGGG) and related motifs *in vitro*. In contrast, Sp2 has largely escaped attention since its initial discovery [16] likely because no DNA-binding activity of full-length Sp2 is detectable by the electrophoretic mobility shift assay [17,18], and because Sp2 has little activation capacity on promoters that are regulated by Sp1 or Sp3 in reporter gene assays [19].

We have recently determined the genome-wide occupancy of Sp2 in mouse embryonic fibroblasts (MEFs) and in HEK293 cells, and have found that Sp2 occupies numerous proximal promoters of essential genes [18]. Bioinformatics analysis of the Sp2 binding sites identified CCAAT- and GC boxes as prevalent motifs in these promoters. On this basis and taking into account the similarity of Sp2 with other Sp factors, we concluded that Sp2 is recruited to its sites in chromatin by binding to the GC box *in vivo*. However, in the global chromatin context, it remains a largely unanswered question whether the very similar transcription factors Sp1, Sp2 and Sp3 are bound to the same promoters *in vivo* and whether regions outside the *bona fide* DNA-binding domain contribute to their binding site selection.

To address this important question, we have compared the genome-wide chromatin occupancy of Sp1, Sp2 and Sp3 with each other in mouse embryonic fibroblasts. We found that Sp1 and Sp3 essentially occupy the same promoters and localize to GC boxes. In marked contrast, Sp2 predominantly localizes at CCAAT motifs. By re-expression of various Sp2 and Sp3 mutants in corresponding Sp2 and Sp3 knockout (*Sp2ko* and *Sp3ko*) MEFs, we found that the zinc finger region mediates chromatin binding of Sp3. Unexpectedly, the *bona fide* zinc finger DNA-binding domain is completely dispensable for binding of Sp2. Rather, it is exclusively the glutamine-rich N-terminal domain, which mediates recruitment of Sp2 to its genomic sites. We further show that Sp2 colocalizes with the trimeric CCAAT-binding transcription factor Nf-y at a large fraction of Sp2 binding sites. We provide evidence that Nf-y is necessary for recruitment of Sp2 and suggest that, in turn, Sp2 potentiates Nf-y binding to shared sites, since binding of Nf-y to sites that are also bound by Sp2 is attenuated in *Sp2ko* MEFs. Therefore, we have discovered that the seemingly similar transcription factors Sp1/Sp3 and Sp2 utilize completely different modes of genomic binding site selection and shed light on the previously enigmatic properties of Sp2.

## Results

### ChIP-seq analysis indicate that Sp1 and Sp3 occupy the same promoters

Recently, we have identified genomic binding sites of the transcription factor Sp2 in MEFs [18]. To identify the binding sites for the related transcription factors Sp1 and Sp3, and to elucidate the potential overlap with Sp2, we performed ChIP-seq analysis with the same cells. Two different antibodies for each factor that do not cross-react with other Sp family members were used ([8,18] and S1–S2 Figs). An Sp3 ChIP using *Sp3ko* MEFs served as a control for the selection of Sp3-specific peaks; and an IgG ChIP for the selection of Sp1-specific peaks, as *Sp1ko* MEFs are not viable.

**Fig 1:**
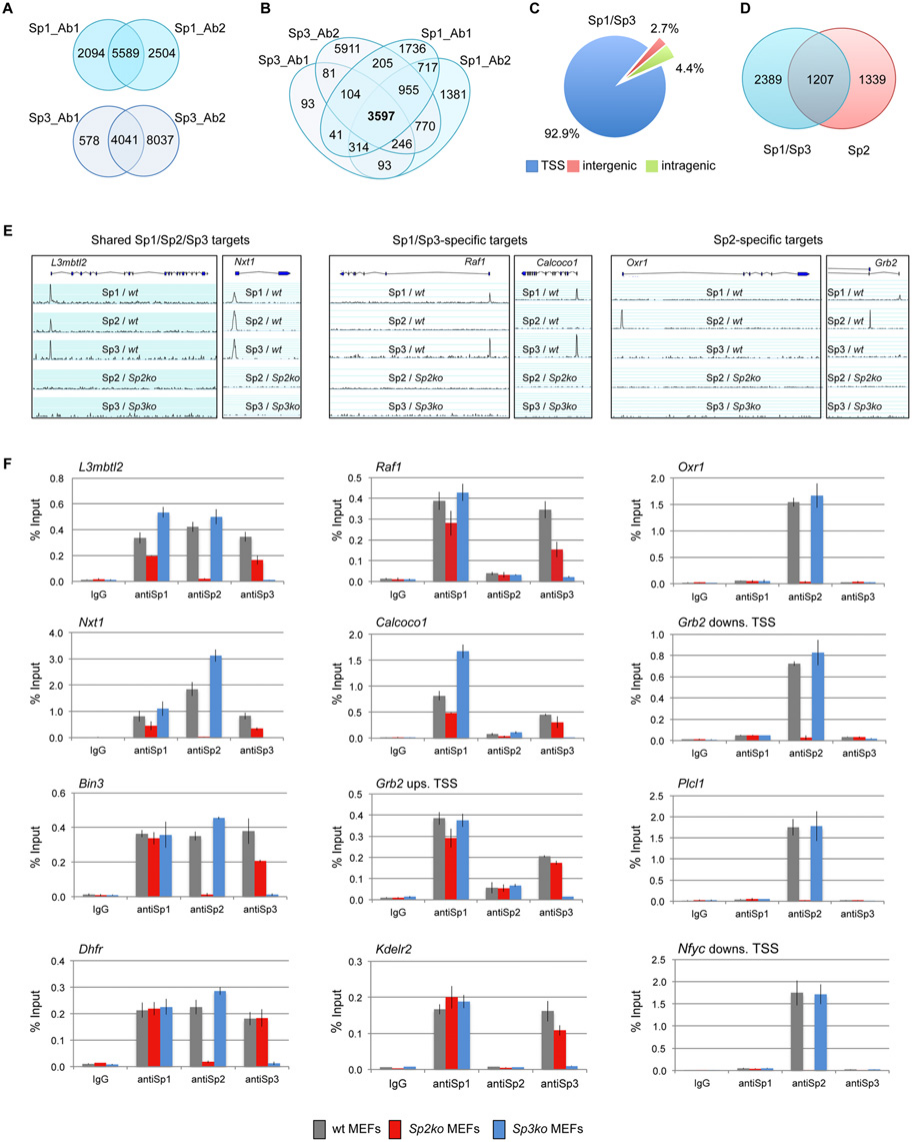
Different binding site selection of Sp1/Sp3 and Sp2 in MEFs. Binding sites of Sp1 and Sp3 in MEFs were determined by ChIP-seq. (A) Venn diagrams showing the overlap of Sp1 or Sp3 peaks obtained with two different antibodies for each factor. (B) Venn diagrams showing the overlap of Sp1 and Sp3 peaks. 3597 overlapping peaks were obtained with all four antibodies. (C) Distribution of Sp1/Sp3 binding sites in MEFs relative to annotated genes (TSS, +/- 500 bp). (D) Overlap of high-confidence Sp1/Sp3 and Sp2 binding sites. (E) Representative genome browser snapshots of promoters bound by all three Sp factors (left), by Sp1 and Sp3 but not by Sp2 (middle), or exclusively by Sp2 (right) in wild type (wt), *Sp2ko* and *Sp3ko* MEFs. (F) ChIP-qPCR validation experiments using Sp1_Ab1, Sp2_Ab1 and Sp3_Ab1. Percent of input values represent the mean of at least three independent experiments +/- SD.

We identified 5589 Sp1 and 4041 Sp3 peaks as overlapping across two sets of samples using two different antibodies for each factor (Fig. 1A). Comparing the Sp1 and Sp3 peaks of all four ChIP-seq data sets revealed 3597 high-confidence sites that are bound by Sp1 as well as by Sp3 (Fig. 1B). The large majority of these sites (~93%) are located close to the 5´-end (+/- 500 bp) of annotated transcripts (Fig. 1C). The comparison of the Sp1- and Sp3 ChIP-seq data sets also revealed a fraction of ~700 Sp1-specific peaks and a few (81) Sp3-specific peaks (Fig. 1B). The majority of these sites represent peaks with relatively low tag counts just above the threshold used for peak selection. Another fraction of the potential Sp1-specific peaks is also found in the Sp3 ChIP-seq data sets. However, these peaks were removed from the classified Sp3 list because a peak appeared also in *Sp3ko* MEFs. The latter observation highlights the great benefit of using knockout cells as ChIP controls. Taken together, we are not convinced about the reliability of the apparently specific Sp1- or Sp3 binding sites. Rather, our ChIP-seq results support the notion that Sp1 and Sp3 essentially occupy the same promoters *in vivo*. Combined binding of Sp1 and Sp3 to the same promoters is consistent with the phenotype of *Sp1/Sp3* compound heterozygous mice. These mice are not viable suggesting that a critical threshold of Sp1 and Sp3 activity is required for normal embryonic development and for proper regulation of common target genes [20].

### Genomic binding of Sp1/Sp3 and Sp2 is different

We next compared the Sp1/Sp3 binding sites with those of Sp2 [18]. Although there is a large overlap, we were able to distinguish, with high confidence, Sp1/Sp3-specific as well as Sp2-specific binding sites (Fig. 1D). Genome browser snapshots of representative shared Sp1/Sp2/Sp3, Sp1/Sp3-specific, and Sp2-specific binding sites are shown in Figs. 1E and S2. We also probed a panel of selected target promoters by conventional ChIP-qPCR analysis, and confirmed binding of all three Sp-factors to common promoters such as the *L3mbtl2*, *Nxt1*, *Bin3* and *Dhfr* promoter, Sp1/Sp3-specific binding to the *Raf1*, *Calcoco1*, *Kdelr2* and the *Grb2* upstream promoter, and Sp2-specific binding to the *Oxr1*, *Plcl1*, and the *Nfyc* and *Grb2* downstream promoters (Fig. 1F), the latter containing alternative promoters that are either occupied by Sp1/Sp3 or Sp2. Of note, consistent with our previous observation [18], Sp1 and Sp3 binding to several promoters is reduced in *Sp2ko* MEFs, which is likely due to their lower expression levels in these cells [18].

### Sp1/Sp3 and Sp2 binding sites show different sequence motif distributions

**Fig 2:**
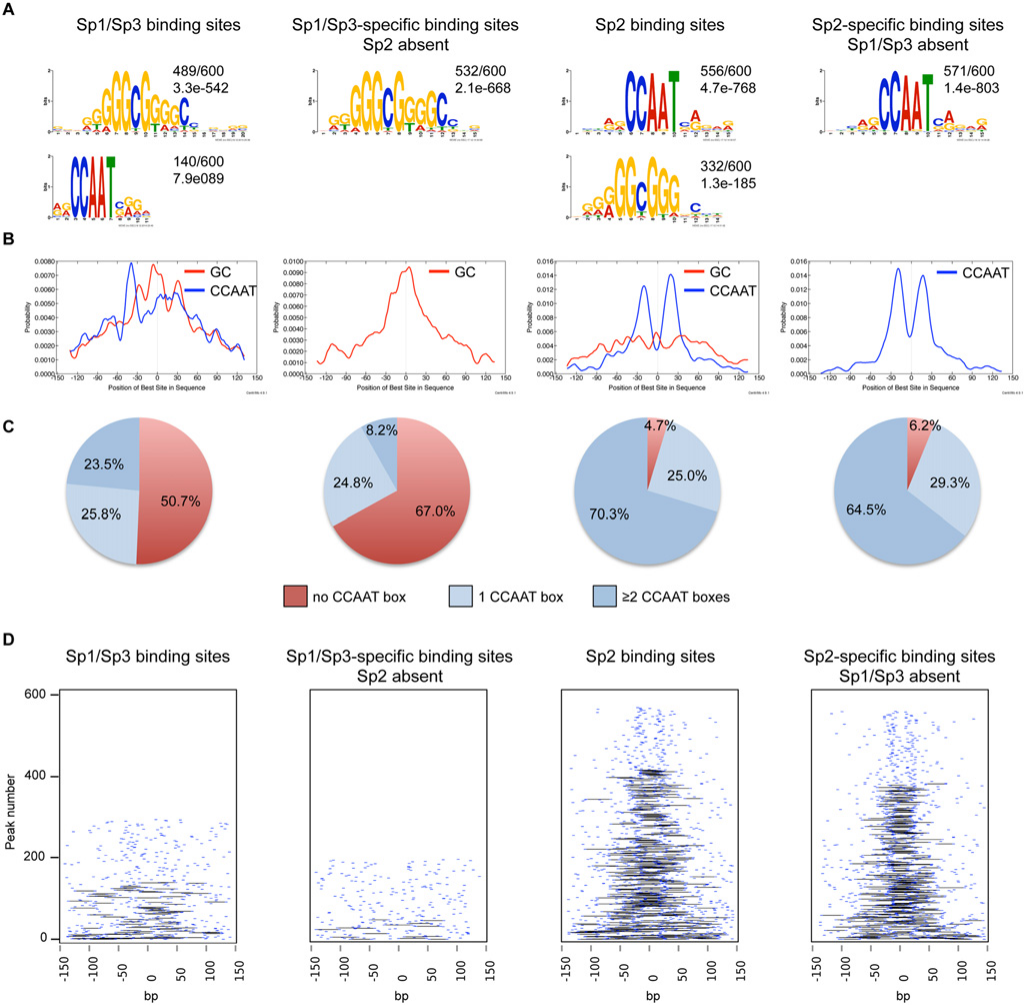
Sequence motifs at Sp1/Sp3 and Sp2 binding sites in MEFs. (A) Sequence motifs enriched at Sp1/Sp3 and Sp2 binding sites. Logos were obtained by running MEME-ChIP [48] with 300 bp summits of the top 600 Sp1/Sp3, Sp2, Sp1/Sp3-specific and Sp2-specific ChIP-seq peaks. The numbers next to the logos indicate the occurrence of the motifs, and the statistical significance (*E*-value). (B) Central motif enrichment analysis of the motifs shown in Fig. 2A. (C) Occurrence of the canonical “CCAAT” sequence at the top 600 Sp1/Sp3, Sp2, Sp1/Sp3-specific and Sp2-specific ChIP-seq peaks. (D) Pairwise distribution of the canonical “CCAAT” sequence at the top 600 Sp1/Sp3, Sp2, Sp1/Sp3-specific and Sp2-specific ChIP-seq peaks. Each short blue line indicates a CCAAT motif. Black lines connect two CCAAT motifs in a promoter if they are located within a distance of 30 to 50 nucleotides.

We performed an unsupervised *de novo* sequence motif analysis at Sp1/Sp3 and Sp2 binding sites. The top motif at the Sp1/Sp3 peaks matches well-known *in vitro* Sp1/Sp3 binding sites with the GGGCGGG core sequence (GC box). The second enriched motif is “CCAAT”, which is a binding site for the transcription factor Nf-y. Essentially, the same two motifs are found at the Sp2 binding sites [18]. However, at the Sp2 binding sites, the CCAAT motif is much more prevalent than the GC box motif (Fig. 2A). Moreover, only the GC box motif but not the CCAAT motif is enriched at sites that are bound by Sp1 and Sp3 but not by Sp2. Conversely, only the CCAAT motif but not the GC box is enriched at sites that are bound by Sp2 but not by Sp1 and Sp3 (Fig. 2A). To extract positional information for the GC and CCAAT motifs within Sp1/Sp3 and Sp2 peaks, we performed a central motif enrichment analysis (CMEA) [21]. This analysis revealed that the GC box motif is enriched at the peak centers of the Sp1/Sp3 binding sites, whereas the CCAAT box motif is found at flanking regions showing a multimodal shape (Fig. 2B). A strikingly different picture emerges at the Sp2 binding sites. Most significantly, the GC box motif is barely enriched at the Sp2 peak centers, whereas the CCAAT box motif exhibits a centrally enriched, symmetrical bimodal distribution with a mean distance of ~35 bp (Fig. 2B).

Intrigued by the nicely shaped distribution of the CCAAT motifs at the Sp2 binding sites, we examined the distribution of the CCAAT motif in greater detail. We found that ~70% of the top Sp2 binding sites but only ~23% of the Sp1/Sp3 sites contain two, or more, perfect CCAAT motifs. Moreover, ~65% of the Sp2-specific sites but only ~8% of the Sp1/Sp3-specific sites contain more than one CCAAT motif (Fig. 2C). Finally, many Sp2-specific promoters (~40%) but only a few Sp1/3-specific promoters (2.3%) contain two CCAAT/ATTGG motifs that are located within a distance of 30 to 50 nucleotides (Fig. 2D). Thus it appears that a large fraction of the Sp2 binding sites is characterized by tandem CCAAT motifs. The CCAAT motif is a binding site for the transcription factor Nf-y. Since Nf-y is also found at promoters that contain imperfect CCAAT motifs [22], the number of Sp2 binding sites with tandem arranged CCAAT motifs (e.g. Nf-y binding sites, see below) could be even higher.

Finally, we also determined the binding sites of Sp1 in HEK293 cells and compared the motifs at the Sp1 binding sites with those at the Sp2 binding sites [18]. Similar to mouse cells, the GC box is the prevalent motif at the Sp1 binding sites, whereas the CCAAT box is the prevalent motif at the Sp2 binding sites (S3 Fig). In summary, the comparison of the genomic Sp1/Sp3 and Sp2 binding sites revealed markedly different sequence motif distributions indicating that binding of Sp2 to chromatin is distinct from Sp1/Sp3, and questions our previous conclusion that Sp2 is recruited to its target promoters *in vivo* via binding to the GC box [18].

### The Sp2 DNA-binding domain is dispensable for genomic binding

Intrigued by the finding that Sp1 and Sp3 are present at GC boxes, whereas Sp2 is located primarily at CCAAT motifs, we sought to identify the protein domains that are responsible for the differences in binding site selection *in vivo*. We focused on Sp2 and Sp3 because corresponding knockout MEFs are available [7,23] that have been proven to be useful for rescue experiments [18,24,25].

**Fig 3:**
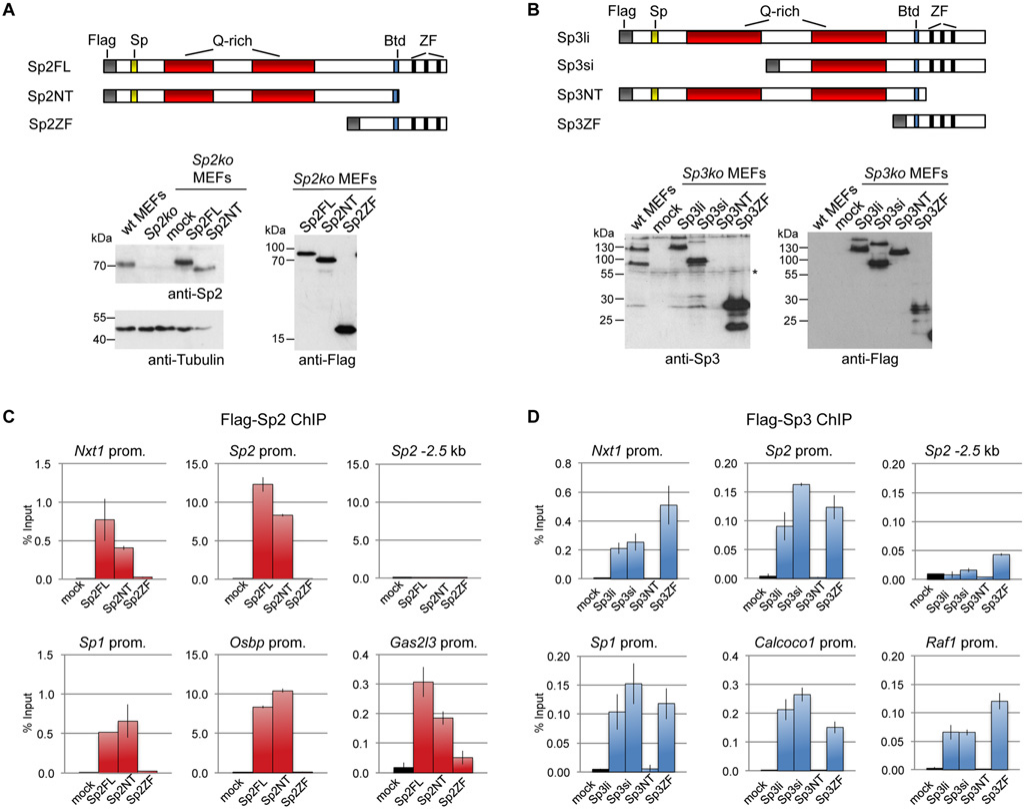
Different domains mediate recruitment of Sp2 and Sp3 to promoters *in vivo*. Flag-tagged full-length Sp2 (Sp2FL) and Sp3 (Sp3li or Sp3si, long or short isoforms) and deletion mutants encompassing either the N-terminal domains (Sp2NT and Sp3NT) or the C-terminal zinc finger domains (Sp2ZF and Sp3ZF) were re-expressed in corresponding *Sp2ko* or *Sp3ko* MEFs. (A, B) Top panels, schematic representation of the Sp2 and Sp3 mutants. The Flag-epitope at the N-termini (grey), the Sp box (yellow), the glutamine-rich domains (Q-rich, red), the Btd box (blue) and the zinc fingers (ZF, black bars) are indicated. Bottom panels, expression of the Sp2 and Sp3 mutants was monitored by immunoblotting using anti-Sp2, anti-Flag and anti-Sp3 antibodies as indicated. Re-probing for tubulin controlled loading of Sp2-containing extracts. The non-specific band indicated by an asterisk served as loading control for Sp3-containing extracts. Of note, the anti-Sp3 antibody does not recognize the Sp3NT fragment because it binds to a C-terminal epitope. (C, D) Binding of Sp2 and Sp3 mutants to selected target promoters was analyzed by ChIP-qPCR. Anti-Flag antibodies were used for ChIP. The percent of input values are mean +/- SD (n = 3).

We stably expressed Flag-tagged full-length Sp2 and Sp3, and deletion mutants thereof in corresponding knockout MEFs. All proteins are expressed at levels similar to endogenous Sp2 and Sp3 with the exception of the zinc finger domains that are expressed at a higher level (Fig. 3A-B). ChIP-qPCR analysis of selected target promoters including promoters that are bound by Sp2 as well as by Sp3 (*Nxt1*, *Sp1* and *Sp2*), and promoters that are preferentially bound by either Sp2 (*Gas2l3* and *Osbp*) or Sp3 (*Calcoco1* and *Raf1*) revealed specific binding of re-expressed full-length Sp2 and the full-length Sp3 isoforms (Sp3li and Sp3si) (Fig. 3C-D). The Sp3ZF fragment lacking the entire N-terminal part is also bound at the Sp3 target promoters showing that the Sp3 zinc finger domain is sufficient for binding. As expected, the Sp3NT mutant lacking the DNA-binding domain is not bound at any of these promoters (Fig. 3D). The picture, which emerged from the analysis of the Sp2 mutants is completely different. The Sp2ZF fragment lacking the N-terminal part is not bound at any of the Sp2 target promoters (Fig. 3C). Strikingly, the Sp2NT mutant expressing the entire N-terminal part but lacking all three zinc fingers is bound at the *Nxt1*, *Sp1*, *Sp2*, *Gas2l3* and *Osbp* promoters almost as strongly as full-length Sp2 (Fig. 3C). Thus, the canonical DNA-binding domain of Sp2 is dispensable, and the N-terminal part sufficient for binding to these promoters *in vivo*. This result suggests that Sp2 is recruited to its target promoters indirectly, likely involving protein-protein interactions (see Discussion chapter for further details).

**Fig 4:**
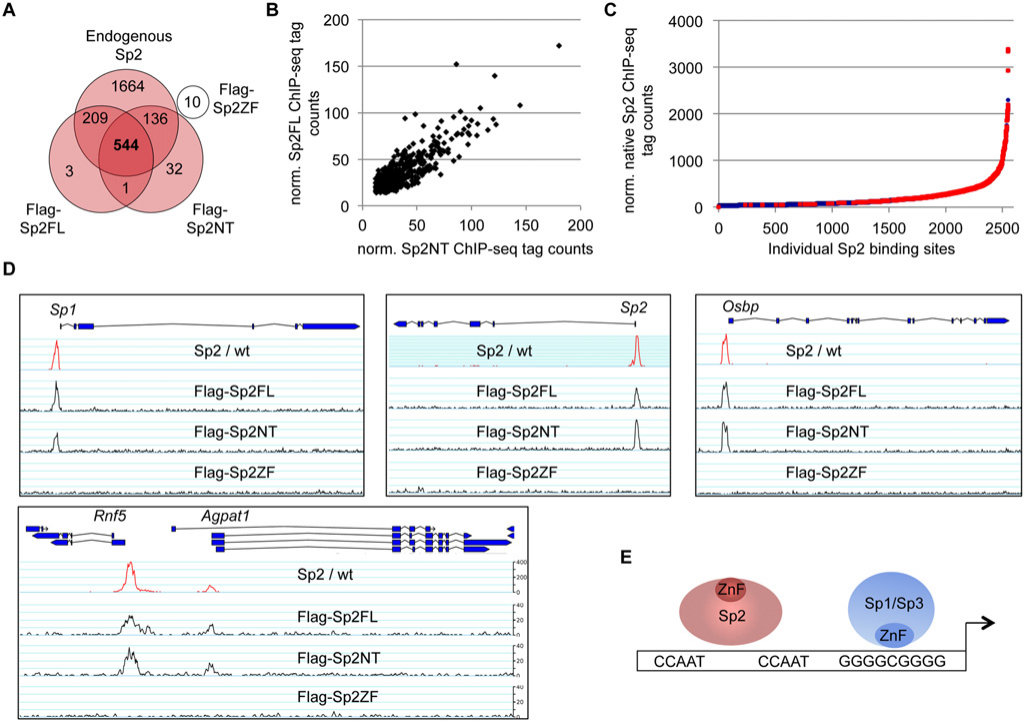
The *bona fide* DNA-binding domain of Sp2 is dispensable for genomic binding. *Sp2ko* MEFs re-expressing Flag-tagged full-length Sp2 (Flag-Sp2FL), the N-terminal domain (Flag-Sp2NT) or the C-terminal zinc finger domains (Flag-Sp2ZF) were subjected to ChIP-seq analysis. (A) Venn diagram showing the overlap of sites bound by Flag-Sp2FL, Flag-Sp2NT and Flag-Sp2ZF with sites bound by endogenous Sp2 in wild type MEFs. (B) Full-length Sp2 and the N-terminal region of Sp2 have similar chromatin binding efficiencies. ChIP-seq tag counts (normalized to 20x10^6^ reads) at individual Flag-Sp2NT and Flag-Sp2FL peaks found in both samples were plotted against each other. (C) Sites bound by Flag-Sp2NT represent high affinity binding sites of native Sp2. Individual native Sp2 peaks [18] were plotted against their normalized tag counts. Those sites that were also detected by ChIP-seq in *Sp2ko* MEFs expressing the N-terminal domain of Sp2 (Flag-Sp2NT mutant) were overlaid with red dots. (D) Representative binding profiles of Sp2 in wild type MEFs (Sp2 / wt), and of Flag-Sp2FL, Flag-Sp2NT and Flag-Sp2ZF expressed in *Sp2ko* MEFs. (E) Schematic representation of the genomic binding features of Sp1/Sp3 and Sp2 based on the results shown in Figs. 1–4.

To further substantiate the conclusion that Sp2 is recruited to its genomic sites by the N-terminal region rather than by the C-terminal zinc finger domain, we performed ChIP-seq using *Sp2ko* MEFs re-expressing Flag-tagged versions of full-length Sp2 (Sp2FL), the Sp2 N-terminal part (Sp2NT) or the Sp2 zinc finger domain (Sp2ZF; see scheme in Fig. 3A). Although the Flag ChIP was less efficient in this particular experiment, we identified more than seven hundred highly reliable Sp2FL and Sp2NT binding sites but only ten potential Sp2ZF sites (Fig. 4A). Comparison of these binding sites with those of endogenous Sp2 in wild type MEFs revealed that >99% of the Sp2FL and >95% of the Sp2NT sites, but remarkably none of the Sp2ZF sites correspond to native Sp2 binding sites (Fig. 4A). The correlation of normalized tag counts at individual Flag-Sp2FL and Flag-Sp2NT peaks (Fig. 4B) shows that full-length Sp2 and the zinc finger-deficient Sp2NT mutant bind to chromatin with similar strength. Of note, the sites bound by the Sp2NT moiety correspond to the strongest native Sp2 peaks (Fig. 4C). Representative genome browser snapshots of Sp2FL and Sp2NT peaks in comparison with native Sp2 peaks are shown in Fig. 4D. Taken together, these striking results show convincingly that the zinc finger domain is dispensable for genomic binding of Sp2 to its target promoters. Recruitment of Sp2 *in vivo* by its N-terminal region is not limited to just a few selected target sites, but is a general feature of Sp2-targeting to chromatin (Fig. 4E).

**Fig 5:**
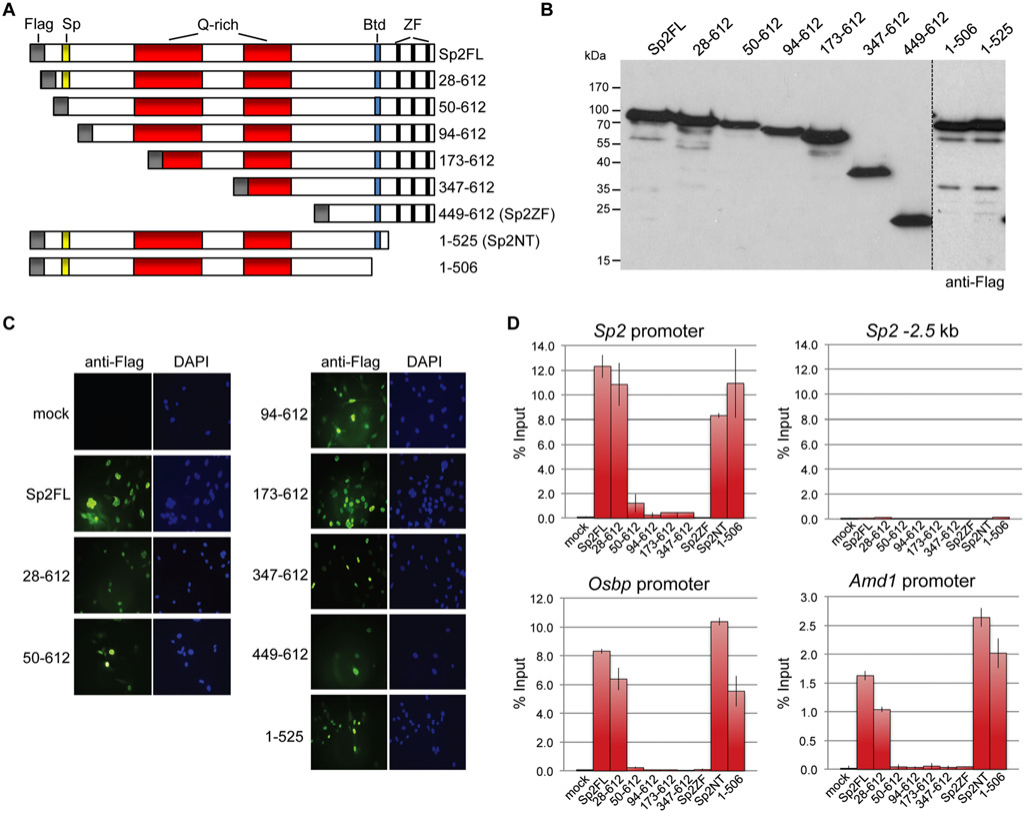
Binding of Sp2 deletion mutants to selected target promoters. (A) Schematic representation of Sp2 deletion mutants expressed in *Sp2ko* MEFs. (B) Western blot analysis (anti-Flag) of the Sp2 mutants shown in panel A. (C) Subcellular localization of the Sp2 mutants in rescued *Sp2ko* MEFs. Sp2 was detected by immunofluorescence using anti-Flag antibodies. Nuclei were counterstained with DAPI. (D) ChIP-qPCR analysis of Sp2 mutants at selected target promoters (*Sp2*, *Osbp* and *Amd1*). The *Sp2–2*.*5* kb region served as a negative control. The percent of input values are mean +/- SD (n = 3).

To map protein regions of Sp2 that are essential for chromatin binding *in vivo*, we stably re-expressed a series of N-terminal Sp2 deletion mutants (Fig. 5A) in *Sp2ko* MEFs and tested their binding to selected loci. Western blotting and immunohistochemistry control experiments showed that all mutants are expressed at similar levels and are present in the nucleus (Fig. 5B-C). Deletion of 27 N-terminal amino acids does not affect binding of Sp2, whereas deletion of 93 N-terminal amino acids completely abolishes binding (Fig. 5D). The Sp2 mutant lacking 49 N-terminal amino acids shows some residual binding to the *Sp2* and *Osbp* promoters. We conclude that the Sp2 sequence between amino acids 28 and 93 is essential for binding of Sp2 *in vivo*. This region contains the Sp-box (33-SPLALLAATCSKIG-46), a hallmark of the Sp transcription factor family members [1]. Whether the Sp-box is directly involved in recruitment of Sp2 remains to be established. Finally, we also rescued *Sp2ko* MEFs with a C-terminal deletion mutant (1–506 mutant in Fig. 5A) that lacks the zinc finger domain and, in addition, the buttonhead box. The buttonhead box is a cysteine-rich motif (CxCPnC) of unknown function, and it is also a hallmark of Sp factors. The Sp2 1–506 mutant binds to the *Sp2*, *Osbp* and *Amd1* promoters as efficiently as full-length Sp2 demonstrating that the buttonhead box is also dispensable for binding of Sp2 to its sites *in vivo*.

### The N-terminal region of Sp2 rescues target gene expression

**Fig 6:**
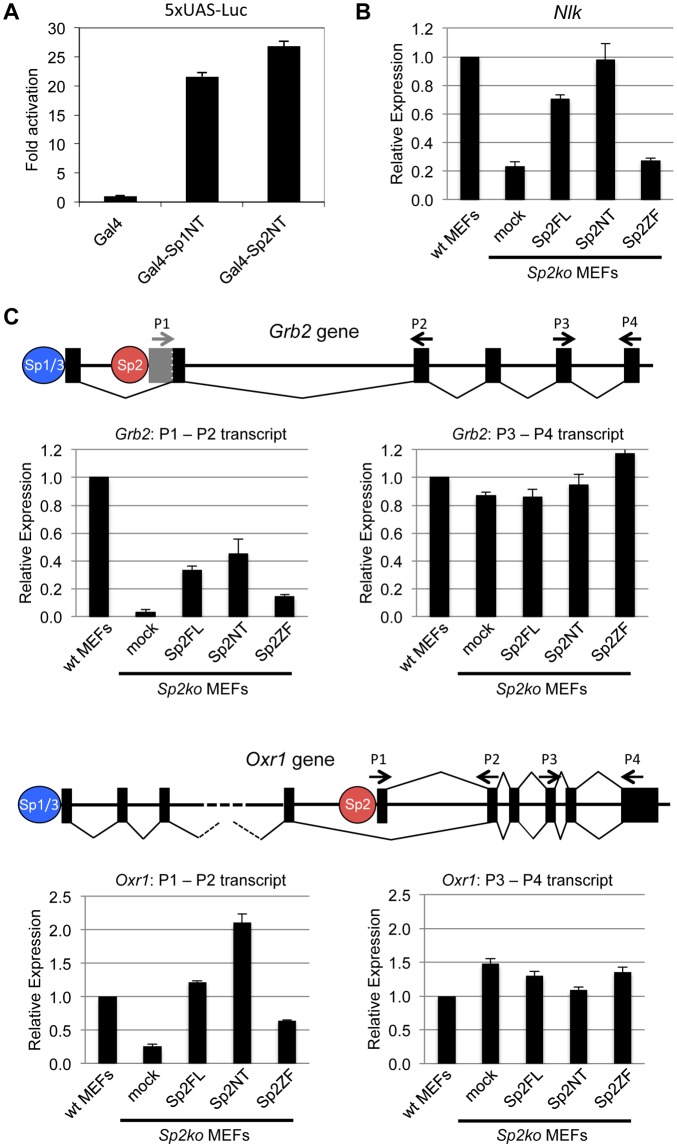
The N-terminal domain of Sp2 can rescue target gene expression. (A) The N-terminal domain of Sp2 has the capacity to activate transcription. The Gal4 DNA-binding domain (Gal4), and Gal4-Sp1NT and Gal4-Sp2NT fusions were transfected into HEK293 cells along with a 5xUAS-luciferase reporter construct. Fold activation by Gal4-Sp1NT and Gal4-Sp2NT is expressed relative to the Gal4 DNA-binding domain set to 1. (B, C) Relative expression of the *Nlk* (B), *Grb2* and *Oxr1* (C) genes in wt MEFs, *Sp2ko* MEFs and in *Sp2ko* MEFs re-expressing full-length Sp2 (Sp2FL) or the Sp2NT and Sp2ZF mutants. (C) Schematic representation of the exon-intron structures of the *Grb2* and *Oxr1* genes and the promoters bound by either Sp1/Sp3 or by Sp2. The primer pairs P1-P2 detect specifically transcripts derived from the Sp2-bound promoters. The primer pairs P3-P4 detect all transcripts. Transcript levels in wt MEFs determined by qPCR were set to 1. *Gapdh* mRNA levels were used for normalization. Data are presented as the average of three independent experiments +/-SD.

We wanted to know whether binding of the Sp2NT fragment could affect expression of target genes. At first, we tested whether it has the capacity to activate transcription. We fused the Sp2NT fragment to the Gal4-DNA-binding domain and performed reporter gene assays. The Gal4-Sp2NT fusion protein activated a Gal4-responsive 5xUAS-luciferase reporter as efficiently as a corresponding Gal4-Sp1NT fusion protein (Fig. 6A) showing that the N-terminal part of Sp2 has an activation function.

Next, we tested expression of a selection of Sp2 target genes in wild type, *Sp2ko*, and *Sp2ko* MEFs re-expressing Sp2 mutants. Compared to wild type MEFs, *Nlk* transcript levels are markedly lower in *Sp2ko* MEFs (20% of wt). Nearly wild type *Nlk* mRNA levels are restored in *Sp2ko* MEFs expressing either full-length Sp2 or the Sp2NT mutant, but not in cells expressing the Sp2ZF mutant (Fig. 6B). This result indicates that the loss of Sp2 leads to downregulation of *Nlk* transcription, and that re-expression of the Sp2 N-terminal fragment is sufficient to rescue *Nlk* expression. Nevertheless, the expression levels of many other Sp2 target genes were not significantly affected in *Sp2ko* MEFs [18]. Inspection of annotated ensemble transcripts revealed that many Sp2 target genes (65%) have alternative transcriptional start sites. Moreover, many of the Sp2 binding sites overlap with Sp1/Sp3 binding sites (Fig. 1). Therefore, we reasoned that alternative initiation sites might hide transcriptional initiation driven by Sp2. We chose the *Grb2* and the *Oxr1* genes to test this possibility. Both genes contain an upstream promoter bound by Sp1 and Sp3 but not by Sp2 and a downstream promoter bound by Sp2 but not by Sp1 and Sp3 (Fig. 6C). We analyzed levels of *Grb2* and *Oxr1* transcripts with primer pairs that detect all transcripts, and with primer pairs that detect specifically the transcripts initiated at the Sp2-specific downstream promoters. Compared to wild type MEFs, the total transcript levels of the *Grb2* and *Oxr1* genes are largely unaffected in *Sp2ko* MEFs. In contrast, the downstream promoter-specific transcript levels are significantly lower in *Sp2ko* MEFs; and they are at least partially restored in *Sp2ko* MEFs expressing full-length Sp2 or the Sp2NT mutant (Fig. 6C). In summary, these results indicate that the N-terminal region of Sp2 is sufficient for activation of a subset of genes. However, they do not exclude that the zinc finger domain of Sp2 is essential for activation of other genes.

### The majority of Sp2 sites are also bound by Nf-y

The CCAAT box is the binding site of the ubiquitous transcription factor nuclear factor y (Nf-y, previously also termed Cbf for CCAAT-binding factor; reviewed in [26]). Nf-y is a heterotrimeric protein composed of the three subunits Nf-ya, Nf-yb and Nf-yc. The Nf-ya subunit confers base-specific recognition of the CCAAT sequence, whereas the basic surface of the histone-fold Nf-yb/Nf-yc dimer makes strong contacts with the negatively charged DNA sugar-phosphate backbone [27]. All three Nf-y subunits are necessary for binding to the CCAAT box. Based on its histone-like properties, Nf-y is considered as an architectural promoter organizer that keeps a promoter free of nucleosomes [27].

**Fig 7:**
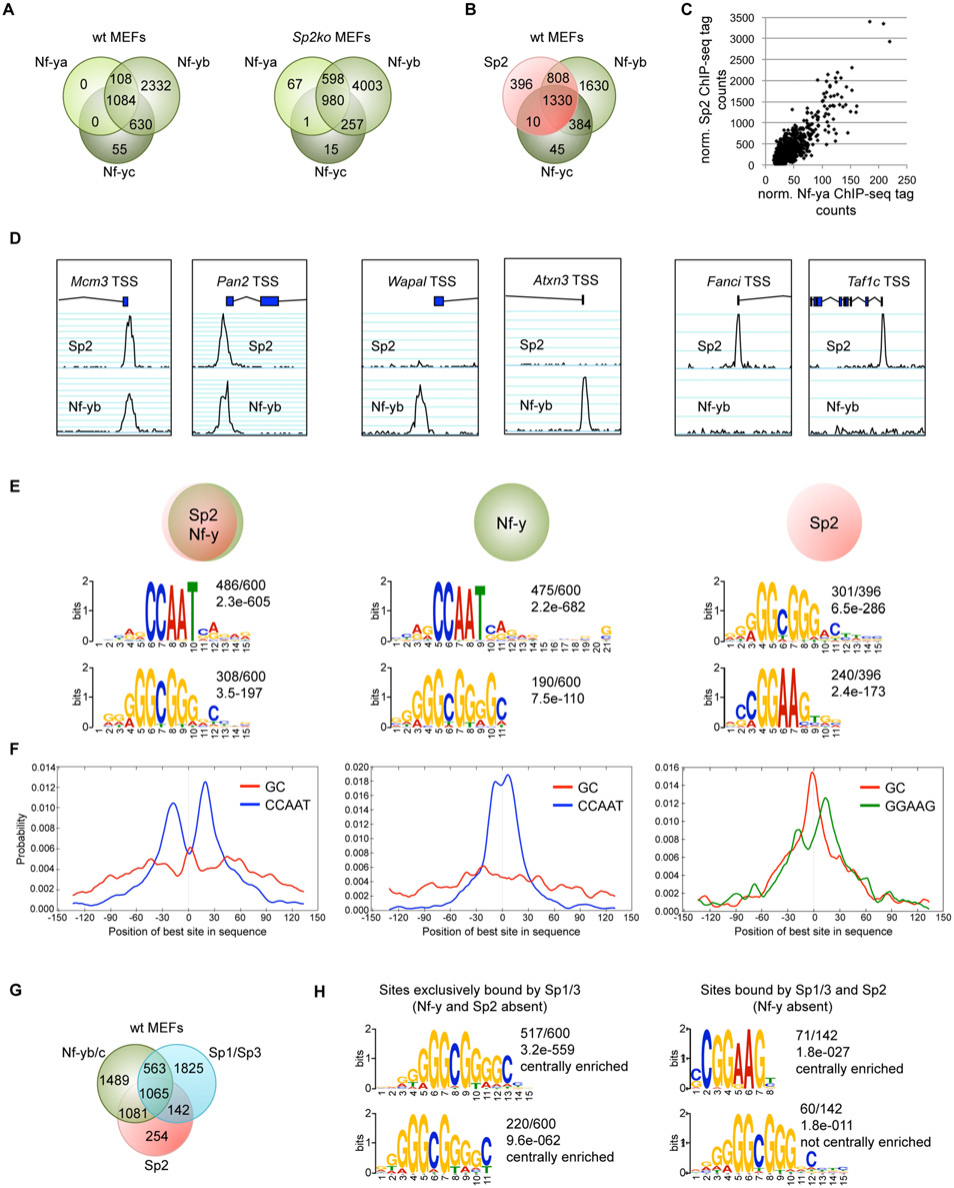
The majority of the Sp2 binding sites are also bound by the heterotrimeric transcription factor Nf-y. Binding sites of the trimeric transcription factor Nf-y were determined by ChIP-seq of the Nf-ya, Nf-yb and Nf-yc subunits using wt and *Sp2ko* MEFs. (A) Venn diagrams showing the overlap of high-confidence Nf-ya, Nf-yb and Nf-yc binding sites in wild type and in *Sp2ko* MEFs. (B) Venn diagram showing the overlap of Nf-y and Sp2 binding sites. (C) The strength of Nf-y binding correlates with the strength of Sp2 binding at shared sites. Normalized ChIP-seq tag counts at individual Nf-ya and Sp2 peaks were plotted against each other. (D) Representative genome browser snapshots of promoters bound by Sp2 as well as by Nf-y (*Mcm3* and *Pan2*), only by Nf-y (*Wapal* and *Atxn3*) or only by Sp2 (*Fanci* and *Taf1c*). (E) Sequence motifs enriched at Sp2 sites also bound by Nf-y (left), at sites only bound by Nf-y (middle) or only bound by Sp2 (right). The numbers next to the logos indicate the occurrence of the motifs, and the statistical significance (*E*-value) [48]. (F) Central motif enrichment analysis of the motifs shown in Fig. 7E. (G) Venn diagram showing the overlap of Sp1/3, Nf-y and Sp2 binding sites. (H) Left, sequence motifs at sites that are bound by Sp1/3 but not by Sp2 and Nf-y. Right, sequence motifs at sites that are bound by Sp2 and Sp1/3 but not by Nf-y.

The presence of Sp2 at CCAAT boxes rather than at the GC box prompted us to ask whether Nf-y is also present at these sites, and whether genomic binding of Nf-y and Sp2 impinge on each other. We performed ChIP-seq of Nf-ya, Nf-yb and Nf-yc with chromatin from wild type and *Sp2ko* MEFs (Fig. 7A). We obtained a higher number of Nf-yb sites with respect to Nf-ya and Nf-yc sites, which very likely does not reflect the occurrence of Nf-yb-specific target loci, but a better ChIP efficiency of the Nf-yb antibody. This assumption is supported by the significantly higher average reads per peak obtained for Nf-yb as well as by the higher enrichment values obtained in ChIP-qPCR experiments (see below). Comparison of the Sp2 and the Nf-y ChIP-seq data sets revealed that 84% of the Sp2 target sites are also bound by Nf-y (Fig. 7B). Moreover, the strength of Nf-y- and Sp2 binding correlates with each other; in other words, sites with high Nf-y subunit tag counts have also high Sp2 tag counts (Fig. 7C). Nevertheless, a subset of sites is clearly bound exclusively by Sp2 or exclusively by Nf-y. Examples of such sites are the *Fanci* and the *Taf1c* promoters that are bound by Sp2 but not by Nf-y, and the *Atxn3* and the *Wapal* promoters that are bound by Nf-y but not by Sp2 (Fig. 7D).

We compared sites that are bound by Sp2 as well as by Nf-y with sites that are bound only by Sp2 or only by Nf-y. MEME reported the CCAAT and the GC motif as the top motifs at sites that are co-bound by Sp2 and Nf-y as well as at sites that are bound by Nf-y but not by Sp2 (Fig. 7E). Consistent with the analysis of all Sp2 ChIP-seq peaks, the CCAAT motif shows a symmetric bimodal distribution at shared Sp2/Nf-y sites. The CCAAT motif at the Nf-y-specific sites does not show this pronounced bimodal distribution but is largely centrally enriched (Fig. 7F). The prevalent motifs at sites bound by Sp2 but not by Nf-y are the GGAAG motif, a binding site for the Ets family members Gabp and Elk4, and the GC box (Fig. 7E). At these sites, the GC box appears to be centrally enriched and flanked by the GGAAG motif (Fig. 7F).

The occurrence of a centrally enriched GC box made it possible that the zinc finger domain of Sp2 could be essential for binding to these sites. Notably, these sites are weak binding sites (S4 Fig) that were not detected in our Flag-Sp2FL and Flag-Sp2NT ChIP-seq experiments shown in Fig. 4. Therefore, we tested binding of Sp2 mutants to this class of promoters by conventional ChIP-qPCR. Full-length Sp2 as well as the Sp2NT fragment are bound to the *Fanci* and the *Taf1c* promoters (S4 Fig). We conclude that the zinc finger region of Sp2 is also dispensable for binding to sites that are not bound by Nf-y.

Finally, we determined the overlap of Sp1/Sp3, Sp2 and Nf-y sites. Approximately 45% of the Sp1/Sp3 sites are also occupied by Nf-y (Fig. 7G). Importantly, the vast majority of the Sp1/Sp3 binding sites that are not bound by Nf-y are also not bound by Sp2 (1825 of 1967; 93%) (Fig. 7G). These sites represent high tag count Sp1/Sp3 binding sites that are enriched of multiple GC boxes and, as expected, do not contain CCAAT motifs (Fig. 7H). Thus, the occurrence of GC boxes and the presence of Sp1/Sp3 are not sufficient to recruit Sp2. Of note, the prevalent, centrally enriched motif at the few Sp2 binding sites that are also bound by Sp1/3 but not by Nf-y (142 sites in Fig. 7G) is the GGAAG motif and not the GC box (Fig. 7H). These findings have also implications concerning the binding of Sp2 to promoters that contain both, GC and CCAAT boxes and are bound by Sp1/Sp3 and by Nf-y (Figs. 2, 7G). At these promoters, the resolution of the ChIP-seq peaks does in most cases not allow to distinguish whether Sp2 is located at a GC box or at a nearby CCAAT box. Given that Sp2 is bound to a large fraction of promoters that are bound by Nf-y lacking Sp1/Sp3 (1081 sites; 42%), it is tempting to conclude that the CCAAT box (i.e. Nf-y) and not the GC box (i.e. Sp1/Sp3) is the decisive sequence for the recruitment of Sp2 to promoters bound by Nf-y and by Sp1/Sp3.

### Sp2 and Nf-y bind simultaneously to shared sites

**Fig 8:**
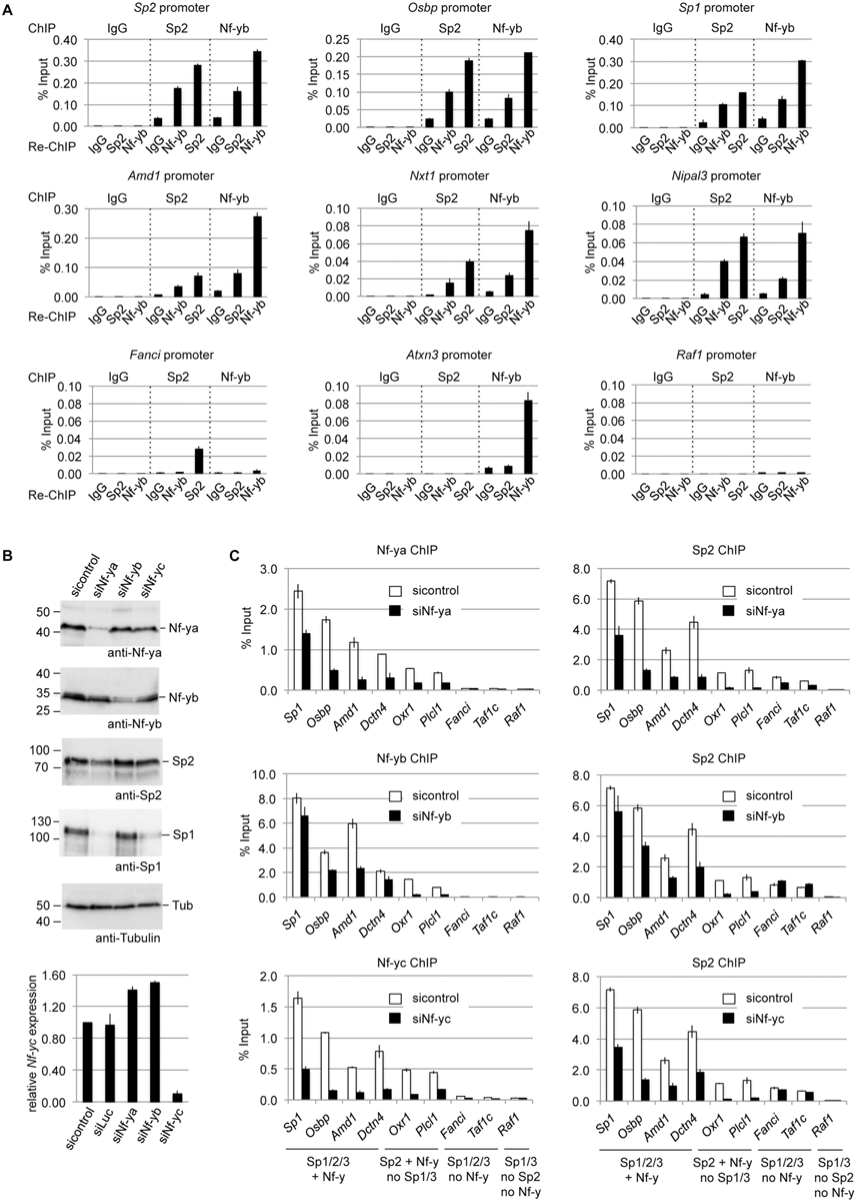
Nf-y is necessary for recruitment of Sp2 to shared sites. (A) Sp2 and Nf-y are bound simultaneously to their target sites. Sp2 and Nf-y occupancy at selected target promoters was analyzed by sequential ChIP (re-ChIP) using anti-Sp2 and anti-Nf-yb antibodies as indicated. The *Sp2*, *Osbp*, *Sp1*, *Amd1*, *Nxt1* and *Nipal3* promoters are co-occupied by Nf-y and Sp2; the *Fanci* promoter is only bound by Sp2 but not by Nf-y; the *Atxn3* promoter is only bound by Nf-y but not by Sp2, and the *Raf1* promoter is neither bound by Sp2 nor by Nf-y. The percent of input values are mean +/- SD (n = 3). (B, C) MEFs were treated with a control siRNA (sicontrol) or siRNAs targeting *Nf-ya*, *Nf-yb* or *Nf-yc*. (B) Top panel, immunoblot analysis of Nf-ya and Nf-yb showing the knockdown efficiency. Bottom panel, knockdown of Nf-yc was controlled by RT-qPCR due to the poor performance of the Nf-yc antibody in immunoblots. (C) Binding of Nf-y subunits and Sp2 to selected promoters after siRNA treatment was analyzed by ChIP-qPCR. The *Sp1*, *Osbp*, *Amd1* and *Dctn4* promoters are co-occupied by Nf-y and all three Sp factors; the *Oxr1* and *Plcl1* promoters are bound by Nf-y and Sp2 but not by Sp1 and Sp3; the *Fanci* and *Taf1c* promoters are bound by all three Sp factors but not by Nf-y; and the *Raf1* promoter is bound by Sp1 and Sp3 but neither by Sp2 nor by Nf-y. The percent of input values are mean +/- SD (n = 3).

Having established the overlap of Sp2 and Nf-y target sites, we tested whether Sp2 and Nf-y simultaneously associate with chromatin, and performed sequential ChIP experiments (re-ChIP). The eluate from Sp2 antibody chromatin precipitation was subjected to precipitation with Nf-yb antibodies, and *vice versa*. Re-ChIPs detected all selected promoters bound by Sp2 and Nf-y (*Sp1*, *Sp2*, *Osbp*, *Amd1*, *Nxt1* and *Nipal3*) independently of the immunoprecipitation order (Fig. 8A), but not the Sp2-specific *Fanci*, the Nf-y-specific *Atxn3* or the Sp1/Sp3-specific *Raf1* promoter. This result demonstrates that Sp2 and Nf-y co-occupy their shared binding sites in the context of chromatin.

### Nf-y is necessary for Sp2 binding

Co-occupancy of Sp2 and Nf-y at shared target promoters led us to ask whether Nf-y is necessary for recruitment of Sp2 to these sites. We knocked down all three Nf-y subunits individually by RNAi (Fig. 8B), and subsequently analyzed binding of Nf-y and Sp2 to a panel of target promoters. These promoters include those that are co-bound by Nf-y and all three Sp factors (*Sp1*, *Osbp*, *Amd1* and *Dcnt4*), promoters that are co-bound by Nf-y and Sp2 but not by Sp1 and Sp3 (*Oxr1* and *Plcl1*), and promoters that are bound by all three Sp factors but not by Nf-y (*Fanci* and *Taf1c*) (Fig. 8C). Lower expression of any of the Nf-y subunits resulted in attenuated binding of Sp2 to all promoters that are co-bound by Nf-y and Sp2. Importantly, the reduction of Sp2 binding is particularly strong at the *Plcl1* and *Oxr1* promoters, which are not bound by Sp1 and Sp3. This result strongly suggests that reduced binding of Sp2 is not an indirect effect of reduced Sp1 levels in Nf-ya and Nf-yc depleted cells (Fig. 8B, panel 4, lanes 2 and 4) but directly caused by attenuated Nf-y binding. Binding of Sp2 to the *Fanci* and *Taf1c* promoters, which are not co-bound by Nf-y, was not affected upon depletion of Nf-yb and Nf-yc. Reduced binding of Sp2 to these two promoters upon Nf-ya knockdown is likely due to lower Sp2 levels in Nf-ya knockdown cells (see Fig. 8B, panel 3, lane 2). Taken together, these results strongly suggest that the presence of Nf-y is necessary for recruitment of Sp2 to shared sites.

### Sp2 potentiates binding of Nf-y

**Fig 9:**
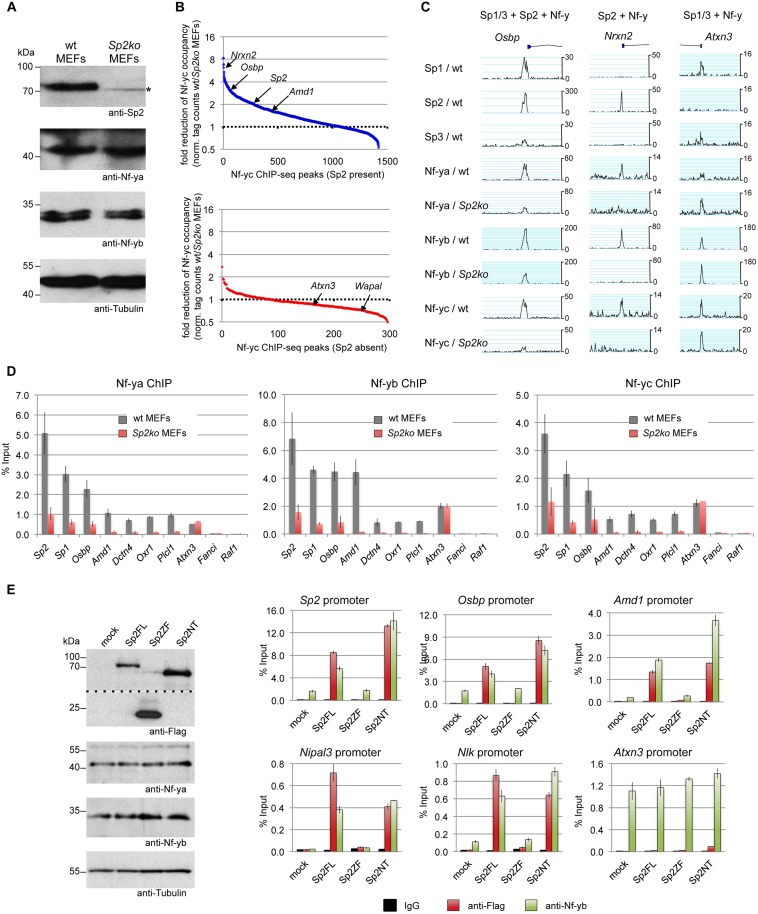
Sp2 potentiates binding of Nf-y at shared sites. (A, B, C, D) Nf-y binding is attenuated in *Sp2ko* MEFs. (A) Western blot analysis of Sp2, Nf-ya and Nf-yb in wild type and in *Sp2ko* MEFs. The asterisk in the Sp2 blot marks a non-specific band. The anti-Tubulin blot served as a loading control. (B) Binding of Nf-y at sites that are also bound by Sp2 is weakened in *Sp2ko* MEFs. Individual Nf-y binding sites were plotted against the ratio (wt/*Sp2ko* MEFs) of normalized Nf-yc tag counts. Top panel, blue dots: High confidence Nf-y binding sites that are co-bound by Sp2 (see Fig. 7B). Bottom panel, red dots: High confidence Nf-y binding sites that are not co-bound by Sp2. For clarity, a subset of promoters (*Nrxn2*, *Osbp*, *Sp2*, *Amd1*, *Atxn3* and *Wapal*) is indicated. (C) Representative binding profiles of Sp1, Sp2, Sp3, Nf-ya, Nf-yb and Nf-yc in wild type and in *Sp2ko* MEFs at a promoter bound by all three Sp factors and Nf-y (*Osbp*), at a promoter bound by Sp2 and Nf-y but not by Sp1/Sp3 (*Nrxn2*), and at a promoter bound by Sp1/Sp3 and Nf-y but not by Sp2 (*Atxn3*). Additional genome browser snapshots are shown in S5–S6 Figs. (D) ChIP-qPCR validations of Nf-y occupancy at selected target promoters in wild type and in *Sp2ko* MEFs (Nf-ya, Nf-yb and Nf-yc ChIPs). The *Sp2*, *Sp1*, *Osbp*, *Amd1* and *Dctn4* promoters are bound by all three Sp factors and by Nf-y; the *Oxr1* and *Plcl1* promoters are bound by Sp2 and Nf-y but not by Sp1/3; the *Atxn3* promoter is bound by Sp1/3 and Nf-y but not by Sp2; the *Fanci* promoter is bound by all three Sp factors but not by Nf-y; and the *Raf1* promoter is bound by Sp1/Sp3 but neither by Sp2 nor by Nf-y. Data are mean of at least three independent experiments +/- SD. (E) Re-expression of Sp2FL or Sp2NT in *Sp2ko* MEFs potentiates Nf-y binding. Left, Western blot analysis of Flag-Sp2 mutants, Nf-ya and Nf-yb in rescued *Sp2ko* MEFs. The anti-Flag blot was split in an upper and a lower part for space reasons. Right, ChIP-qPCR analysis of Flag-Sp2 and Nf-yb binding to selected promoters in rescued MEFs.

Given the dependency of Sp2 on Nf-y, we wanted to know whether Sp2 does affect binding of Nf-y at these shared sites. We compared occupancy of Nf-y in wild type MEFs with occupancy in *Sp2ko* MEFs. Expression of Nf-y is similar in both cell types (Fig. 9A). By calculating the ratio of normalized Nf-y ChIP-seq tag counts in wt and in *Sp2ko* MEFs (wt/*Sp2ko*), we found attenuated Nf-y occupancy in S*p2ko* MEFs at many promoters that are co-bound by Sp2 in wild type MEFs (Fig. 9B, top panel). Attenuated Nf-y binding in *Sp2ko* cells is particularly strong at promoters that are bound in wild type MEFs by Sp2 but not by Sp1/3 such as the *Nrxn2*, *Grb2*, *Nipal3*, *Plcl1* or the *Oxr1* promoters (Figs. 9C-D, S5). This demonstrates that binding of Nf-y to these promoters depends on the presence of Sp2. Importantly, Nf-y binding remains largely unchanged or is even slightly potentiated at sites that are not co-bound by Sp2 (Fig. 9B, bottom panel) including promoters that are also bound by Sp1/Sp3 such as the *Atxn3*, *Mxi1*, and *Pdcd4* promoters (Figs. 9C-D, S6).

Given that genomic Nf-y binding is attenuated in *Sp2ko* MEFs at loci that are co-bound by Sp2, it is to be expected that re-expression of Sp2 potentiates Nf-y binding at these sites. Indeed, we found stronger Nf-y binding in rescued MEFs, specifically at loci that are re-occupied by Sp2FL or by Sp2NT (*Sp2*, *Osbp*, *Amd1*, *Nipal3* and *Nlk*) (Fig. 9E). Binding of Nf-y at these promoters remained unchanged in cells expressing the Sp2ZF fragment. Finally, binding of Nf-y to the *Atnx3* promoter, which is not an Sp2 target, is similar in Sp2FL, Sp2NT and Sp2ZF expressing cells (Fig. 9E). Taken together, these results strongly support the conclusion that Sp2 potentiates binding of Nf-y at shared sites.

## Discussion

In this study, we have elucidated the mode of genomic binding site selection of the zinc finger transcription factors Sp1, Sp2 and Sp3. A key finding is that Sp1/3 and Sp2 bind to chromatin by distinct mechanisms. Consistent with numerous *in vitro* DNA-binding studies, Sp1 and Sp3 localize to GC boxes and essentially occupy the same promoters. Unexpectedly, Sp2 primarily localizes at CCAAT motifs, often arranged in tandem with a mean distance of 35 bp (Fig. 2). In line with their different binding site selection, re-expression of Sp2 and Sp3 mutants in corresponding *Sp2ko* and *Sp3ko* MEFs revealed that different protein domains mediate binding to their sites in chromatin. As expected, the C-terminal zinc finger domain is essential and largely sufficient for binding of Sp3 to its target promoters. Although not formally tested, we consider it as very likely that the zinc finger domain of Sp1 also mediates binding to chromatin. Intriguingly, the zinc finger domain of Sp2 is fully dispensable for binding *in vivo*. Instead, binding of Sp2 to its target promoters is mediated by the N-terminal glutamine-rich part, providing the essential clue for the different binding site selection of Sp2, as compared to Sp1 and Sp3.

Interestingly, *Sp2* targeted mice that do not express the zinc finger domain but aberrantly express an RNA encoding the N-terminal region display a markedly less severe phenotype than *Sp2null* mice [7]. Although expression remains to be established at the protein level, binding of the Sp2 N-terminal region to chromatin shown in this report provides a rational explanation for the different phenotypes of these mice.

Sequence comparisons of the N-terminal region of Sp2 with the corresponding regions of Sp1 and Sp3 reveal major differences of their amino acid composition. Most significantly, in addition to the frequent occurrence of glutamine residues, the N-terminal domains of Sp1 and Sp3 are rich in acidic amino acids, whereas the N-terminal domain of Sp2 is very rich in basic amino acids (S7 Fig). Thus, the general assignment of the N-terminal domains of Sp1, Sp2 and Sp3 as glutamine-rich domains is misleading. Importantly, the presence of multiple positive charged lysines in the N-terminal region of Sp2 raises the possibility of non-specific interactions with the negatively charged sugar-phosphate backbone of the DNA.

Binding sites of Sp1 and Sp2 have also been identified in K562 cells by the ENCODE consortium [28]. Since Sp1 and Sp2 binding sites largely overlap, it was concluded that Sp1 and Sp2 binding motifs (i.e. GC boxes) are indistinguishable [29]. We believe that this interpretation of the ChIP-seq data results from the structural similarity of Sp1 and Sp2, and from Sp2´s capacity to bind GC rich oligonucleotides *in vitro* [18,19]. Admittedly, we also fell into this trap when we initially analyzed our Sp2 ChIP-seq data [18]. These misinterpretations emphasize the limitations of genomics data integration and highlight the importance of performing additional detailed experimental analysis.

The data presented here revealed fundamental different modes of binding site selection of Sp1/Sp3 and of Sp2, and also allow different interpretations of the Sp1-Nf-y and Sp2-Nf-y interactions. Sp1 and Sp3 ChIP-seq peaks locate to GC boxes that are often accompanied by Nf-y binding sites (see Fig. 2) strongly suggesting that Sp1/Sp3 and Nf-y co-bind to neighboring sites in the genome. This is consistent with earlier reports showing synergistic activation by Sp1 and Nf-y and a direct interaction between Sp1 (and Sp3) and Nf-ya [30,31,32]. In contrast, the majority of Sp2 locates at CCAAT boxes and binding is independent of the presence of a nearby GC box, and, most importantly, independent of the zinc finger domain.

The localization of Sp2 at CCAAT motifs led us to detail the interaction network between Sp2 and Nf-y on a genome-wide scale. The arrangement of CCAAT motifs in regions that are bound by both factors differ from sites that are bound by Nf-y but not by Sp2. Sp2-Nf-y interactions occur predominantly at tandem CCAAT boxes, whereas single CCCAT boxes are mostly bound by Nf-y but not by Sp2. Utilizing *Sp2ko* cells lacking any Sp2 DNA-binding activity, and an RNAi approach to interfere with Nf-y binding, we have explored the interaction between Nf-y and Sp2 recruitment in an unbiased manner. Genomic binding analysis revealed a widespread attenuation of Nf-y loading in cells lacking Sp2. *Vice versa*, reduced binding of Nf-y led to attenuation of Sp2 binding. Importantly, the reduction of Sp2 binding is specific to elements that are also bound by Nf-y, thereby providing support for a direct effect of Nf-y at shared sites. Given that multiple CCAAT boxes in a promoter region are simultaneously occupied by Nf-y, the particular placement of Nf-y complexes might be essential for direct or indirect interactions with Sp2. In line with this, we observed a general agreement between the strength of the Sp2 ChIP-seq signals and the number of CCAAT motifs. Particularly, the strongest Sp2 peaks contain several pairs of CCAAT motifs.

In a recent publication it was shown that Nf-y promotes binding of pluripotency transcription factors such as Oct4 or Sox2 at enhancers in murine ES cells by facilitating a permissive chromatin conformation [33]. Nf-y´s role in promoting the binding of Sp2 could be by a similar mechanism. However, unlike the pluripotency factors, Sp2 also potentiates binding of Nf-y at sites of co-localization. Whether Sp2 facilitates recruitment of Nf-y or whether it stabilizes binding of Nf-y at shared sites remains elusive at this stage.

**Fig 10:**
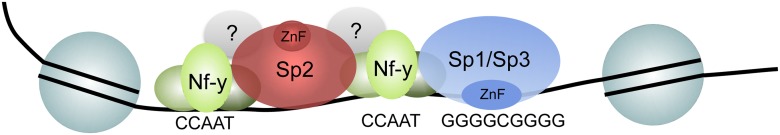
Model depicting the recruitment of Sp1/Sp3 and Sp2 to their target promoters *in vivo*. Consistent with *in vitro* binding data, Sp1 and Sp3 are recruited to GC boxes by their zinc finger domain. The majority of Sp2 is recruited to CCAAT motifs (often arranged in tandem) that are co-bound by Nf-y. Binding of Sp2 to these sites is by its N-terminal region, and is independent of the zinc finger domain. The strength of binding to these sites and the presence of positively charged amino acids in the N-terminal region of Sp2 might indicate that protein-protein as well as unspecific protein-DNA interactions are involved.

The mode of interaction between different transcription factors has been classically categorized into DNA-binding dependent (co-binding) and DNA-binding independent, whereby one transcription factor binds to another that, in turn, binds to DNA (tethering). Tethering to chromatin has been reported for several transcription factors including recruitment of the glucocorticoid receptor by AP1 [34,35] or STAT3 [36] and the estrogen receptor alpha by Runx1 [37]. Likely, tethering mechanisms are also involved in the recruitment of SCL/TAL1 [38] and KLF3 [39] to a subset of their binding sites *in vivo*. The sequence adjacent to CCAAT boxes at which Sp2 is located is highly variable and does not contain a particular sequence motif. Consistently, the N-terminal region of Sp2 does not contain an obvious structural motif that might interact with a specific DNA sequence. It can be proposed that Nf-y binds to the CCAAT box through base-specific contacts. Sp2 would then be recruited by interaction with Nf-y or with additional factors. However, this simple tethering model does not explain the attenuated binding of Nf-y in *Sp2ko* cells at shared sites (Fig. 9), and the exceptionally large Sp2 ChIP-seq peaks and high ChIP-qPCR values (more than 10% of input on highly occupied promoters; see Figs. 3, 5, 9) particularly as formaldehyde preferentially crosslinks proteins to DNA. Therefore, we envisage an alternative mechanism. Potentially, promoters bound by Nf-y, particularly those bound by two or more Nf-y complexes, adopt a particular conformation that allows the basic N-terminal region of Sp2 to interact, directly or indirectly, with two Nf-y complexes simultaneously, and additionally with the DNA backbone in a sequence-independent manner (Fig. 10). Such a scenario would explain the mutual dependency of Sp2 and Nf-y binding to common sites.

Finally, a small fraction of the Sp2 binding sites does not contain CCAAT boxes and are not co-occupied by Nf-y. Nevertheless, binding of Sp2 to these sites is also mediated by the N-terminal region suggesting that binding of Sp2 to these sites is by a similar mechanism. *De novo* motif discovery at these sites revealed enrichment of the Ets transcription factor-binding motif GGAAG. Future work aims to identify the Ets factor that occupies these sites. A promising candidate is the widely expressed heterodimeric Ets transcription factor Gabp [40] that binds as a GABPalpha2beta2 heterotetramer complex to DNA containing two tandem GGAAG sites [41].

In conclusion, the data provided in this report challenge the prevailing view that the transcription factors Sp1/Sp3 and Sp2 regulate transcription by binding to similar promoter elements via their zinc finger domains. Instead, Sp1/Sp3 and Sp2 have distinctive binding landscapes, and their modes of genomic binding site selection are completely different. Collectively, our findings uncover strikingly different recruitment mechanisms of very similar transcription factors, and add another crucial level of detail to the current model of transcription factor binding to chromatin.

## Materials and Methods

### Antibodies

For Western blotting and ChIP of Sp1, Sp2 and Sp3 in MEFs, we used homemade rabbit antibodies [7,42] affinity-purified against the respective recombinant Sp factor. Anti-Sp3 antibodies (Santa Cruz, sc-644) were used for the Western blot shown in Fig. 3B, and anti-Sp2 antibodies (Santa Cruz, sc-643) for ChIP-seq of Sp2 in HEK293 cells. Additional antibodies: Anti-Nf-ya (Santa Cruz, sc-10779), anti-Nf-yb (Genespin, PAb001), anti-Nf-yc (Santa Cruz, sc-7715-R), anti-Flag M2 (Sigma, F3165), anti-Tubulin (Millipore, MAB3408).

### Construction of retroviral vectors and retroviral transduction

Retroviral expression plasmids for 3xFlag-Sp2 and 3xFlag-Sp3 mutants were generated by restriction cloning of PCR fragments into a pBABE3xFlag-puro plasmid. Primer sequences used for PCR can be found in S1 Table. The production of virus stocks, infection of MEFs and the selection of transduced *Sp2ko* and *Sp3ko* MEFs were as described [7].

### Cell growth conditions and generation of cell lines

MEFs were cultured in a 1:1 mixture of Dulbecco’s modified Eagle’s medium—high Glucose (PAA) and HAM’s F-10 (PAA) supplemented with 10% (v/v) fetal calf serum (PAA) and 1% Penicillin-Streptomycin. Wild type and *Sp3ko* MEFs, and MEFs with floxed Sp2 alleles (*Sp2fl/fl*) were isolated from E13.5 embryos using standard methods and subsequently immortalized by serial passages. *Sp2ko* MEFs were obtained by retroviral transduction of pBABE-Cre-neo as described [7]. *Sp2ko* MEFs have severely impaired proliferation rates [7]. Cells that escaped growth inhibition over time were used for rescue experiments.

### Immunohistochemistry

Immunofluorescence and microscopy was performed as described [43]. In brief, 5x10^4^ MEF cells expressing 3xFlag-Sp2 mutants were grown on coverslips in 6-well plates overnight. Cells were fixed in 4% PFA/PBS for 25 min, permeabilized in 0.2% TritonX-100/PBS for 20 min, and blocked with 3% BSA/PBS for 1 hr. Incubation of the anti-Flag M2 antibody (Sigma, F3165, 1:800 dilution) was for 1 hr at RT. Secondary antibody incubation (anti-mouse AlexaFluor568, Invitrogen, A10037, 1:500 dilution) was performed for 1 hr at RT in the dark. After a final washing step, coverslips were mounted onto glass slides using Vectashield mounting medium with DAPI (Vector Laboratories, Inc.).

### Knockdown of Nf-y subunits

For RNAi-mediated depletion of mouse Nf-ya, Nf-yb and Nf-yc, pools of four On-target plus siRNAs (GE Dharmacon) were used (LU-065522, LU-046072, LU-060374). The siGenome non-targeting siRNA #1 (D-001210–01) was used as unspecific siRNA control. Wild type MEFs on 15 cm plates were transfected with 24 nM siRNA using Oligofectamine (Invitrogen). Three days post-transfection 2x10^6^ cells were replated, and transfected a second time. Additional three days later, cells were collected and cross-linked chromatin was prepared. To monitor knockdown efficiency at the protein level, a chromatin sample was incubated with an equal amount of 2xLaemmli buffer at 100°C for one hour and subsequently analyzed by western blotting. To monitor knockdown efficiency at the transcript level, RNA was isolated using the RNeasy Mini kit (Qiagen) and expression of Nf-ya, Nf-yb and Nf-yc was analyzed by RT-qPCR.

### RT-qPCR

Expression analysis by quantitative RT-qPCR was performed as described in [44] using gene-specific primers (S1 Table).

### ChIP-qPCR

ChIP experiments were performed as described [18] using the One Day ChIP kit (Diagenode). For a sequential ChIP of Sp2 and Nf-yb, the precipitated material of a standard ChIP was eluted twice from the beads with 100 mM NaHCO_3_, 1% SDS, 10 mM DTT for 30 min at 37°C. Eluates were diluted 1:50 with ChIP buffer and subsequently subjected to a second ChIP in accordance with the One Day ChIP kit manual performing an overnight antibody incubation at 4°C. Enrichment was calculated relative to the input of the first ChIP. Primer sequences for ChIP-qPCRs are listed in S1 Table.

### ChIP-seq and data analysis

For ChIP-seq experiments, four to six individual ChIPs were pooled, and precipitated DNA was purified on QIAquick columns (Qiagen). Five nanograms of DNA were used for indexed next generation sequencing library preparation using the MicroPlex library preparation kit (Diagenode) in accordance with the manufacturer’s instructions. Library purification was performed with AMPure magnetic beads (Beckman Coulter) as described in the MicroPlex kit manual. Libraries were quantified on a Bioanalyzer (Agilent Technologies) and subsequently sequenced on an Illumina HiSeq1500 platform, rapid-run mode, single-read 50 bp (TruSeq Rapid SR Cluster Kit—HS, TruSeq Rapid SBS Kit—HS—50 cycle) according to manufacturer´s instructions. An overview of the various ChIP-seq results is shown in S2 Table.

Raw ChIP-seq data were aligned to the mouse genome assembly mm10 or the human genome assembly hg19 using Subread 1.3.3-p3 [45]. Reads were filtered to have at most 5 mismatches to the reference, indels up to 5 bp and to occur at exactly one position in the genome. Peak calling was performed using MACS 1.4 [46] with default parameters. Raw and normalized (to 1 million uniquely aligned reads) read counts were annotated, and peaks were filtered to have at least 30 raw reads and a signal to background (IgG, *Sp2ko* or *Sp3ko*) normalized read ratio of at least 3. Further analysis of peaks such as association with transcripts in the vicinity, classification of genomic position and Venn diagram generation was performed using a custom Python based pipeline. Genome annotation data from Ensembl revision 74 [47] was used. Position and characteristics of ChIP-seq peaks are listed in S3 Table. Venn diagrams were calculated by building the union of the datasets involved, and assigning each union-peak to a region by requiring at least a one-basepair overlap with the input regions. *De novo* motif search including central motif analysis [21] was performed with MEME-ChIP version 4.9.1 [48] using sequences surrounding peak summits (+/- 150 bp). Elongating reads by 200 bp, and determining the position of highest overlap defined summits.

### Data deposition

Raw sequencing data were deposited at ArrayExpress under accession number E-MTAB-2970.

## Supporting Information

S1 Fig Specificity of the Sp1, Sp2 and Sp3 antibodies in immunoblot experiments. (A) Nuclear extracts of wt, *Sp2ko* and *Sp3ko* MEFs were probed with two different affinity-purified Sp1-, Sp2- and Sp3-specific antibodies. (B) Sp1, Sp2 and Sp3 were expressed in insect S2 cells lacking Sp factors. Subsequently, nuclear extracts were subjected to Western blot analysis using the affinity-purified Sp1-, Sp2- and Sp3-specific antibodies. The asterisks indicate non-specific bands.
(PDF)

S2 Fig Specificity of the Sp1, Sp2 and Sp3 antibodies in ChIP-seq experiments. Shown are genomic binding patterns of Sp1, Sp2 and Sp3 at the *Nxt1*, *Raf1* and *Grb2* gene regions in wt, *Sp2ko* and *Sp3ko* MEFs. All three Sp factors are bound at the *Nxt1* promoter, Sp1 and Sp3 but not Sp2 are bound at the *Raf1* promoter, and Sp2 but not Sp1 and Sp3 is bound at the *Grb2* downstream promoter. Due to the lack of *Sp1ko* MEFs, which are not viable, IgG ChIPs served as controls for the Sp1 ChIPs.
(PDF)

S3 Fig Different binding site selection of human Sp1 and Sp2 in HEK293 cells. Sequence motifs (left) and their distribution (right) at Sp1 and Sp2 peaks in HEK293 cells were obtained by running MEME-ChIP [48] with 300 bp summits of the top 1000 Sp1 and Sp2 ChIP-seq peaks. (A) ChIP-seq with homemade Sp1 antibody 1. (B) ChIP-seq with homemade Sp1 antibody 2. (C) ChIP-seq with homemade Sp2 antibody 1 as published in [18]. (D) ChIP-seq with a commercial Sp2 antibody (Santa Cruz, sc-643). The numbers next to the logos indicate the occurrence of the motif (number of sites contributing to the construction of the motif) and the statistical significance (*E*-value).
(PDF)

S4 Fig The zinc finger domain of Sp2 is also dispensable for binding to genomic regions that are not occupied by Nf-y. (A) Sites of Sp2 that are not co-bound by Nf-y represent low tag count Sp2 binding sites. Individual native Sp2 peaks in wt MEFs [18] were plotted against their normalized tag counts. Those sites that are not co-bound by Nf-y (see Fig. 7B) were overlaid with red dots. (B) Binding of Flag-tagged Sp2FL, Sp2ZF and Sp2NT to the *Fanci* and *Taf1c* promoters was analyzed by ChIP-qPCR. Anti-Flag antibodies were used for ChIP. The percent of input values are mean +/- SD (n = 3).
(PDF)

S5 Fig Genomic binding of Nf-y at the Sp2-specific *Grb2*, *Nipal3* and *Nrnx3* promoters is strongly reduced in *Sp2ko* MEFs. Shown are genome browser snapshots.
(PDF)

S6 Fig Genomic binding of Nf-y at the Sp1/3-specific *Mxi*1, *Atxn3* and *Pdcl4* promoters is not reduced in *Sp2ko* MEFs. Shown are genome browser snapshots.
(PDF)

S7 Fig Amino acid sequences of Sp1, Sp2 and Sp3. The Sp box (yellow), the Btd box (blue) and the zinc finger region (bold) are highlighted. Colored letters within the N-terminal part highlight basic amino acids (K and R, violet), acidic amino acids (D and E, green) and glutamine residues (gray).
(PDF)

S1 Table Sequences of the oligonucleotide primers used for cloning, ChIP-qPCR and RT-PCR analysis. (XLSX)

S2 Table Overview of ChIP-seq results. For each ChIP-seq sample, the number of usable reads, the number of filtered peaks, the average number of tags per peak, and the genomic distribution of the peaks (peaks within 500 bp of TSS, intergenic and intragenic) is shown.
(XLSX)

S3 Table Position and characteristics of ChIP-seq peaks obtained with different cell lines and antibodies as indicated. Each sheet contains the following columns: A, chromosome; B and C, chromosomal region spanning the peak; D, genomic position (TSS, intron, exon or intergenic); E, distance of peak to next TSS; F and G, name and Ensemble ID of the next gene; H, tag counts at individual peaks; I, tag counts normalized to 1 million reads. The sheets are sorted by column I.
(XLSX)
