# Supplementary figures and images for: Zinc Finger Independent Genome-Wide Binding of Sp2 Potentiates Recruitment of Histone-Fold Protein Nf-y Distinguishing It from Sp1 and Sp3
Source: PLoS Genet. 2015 Mar 20;11(3):e1005102. doi: 10.1371/journal.pgen.1005102 (PMC4368557; doi:10.1371/journal.pgen.1005102)

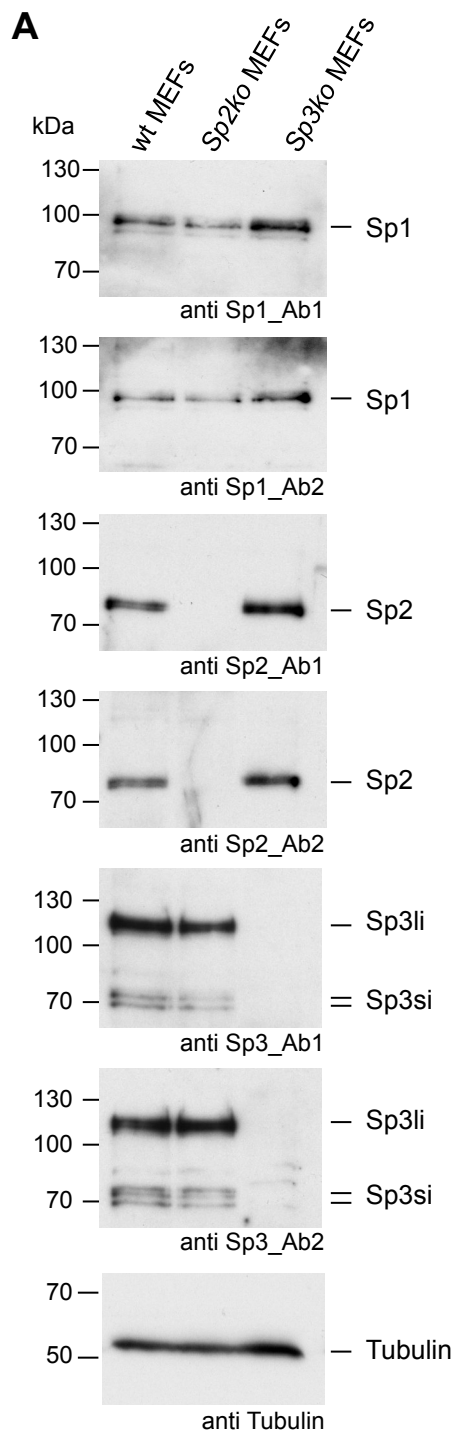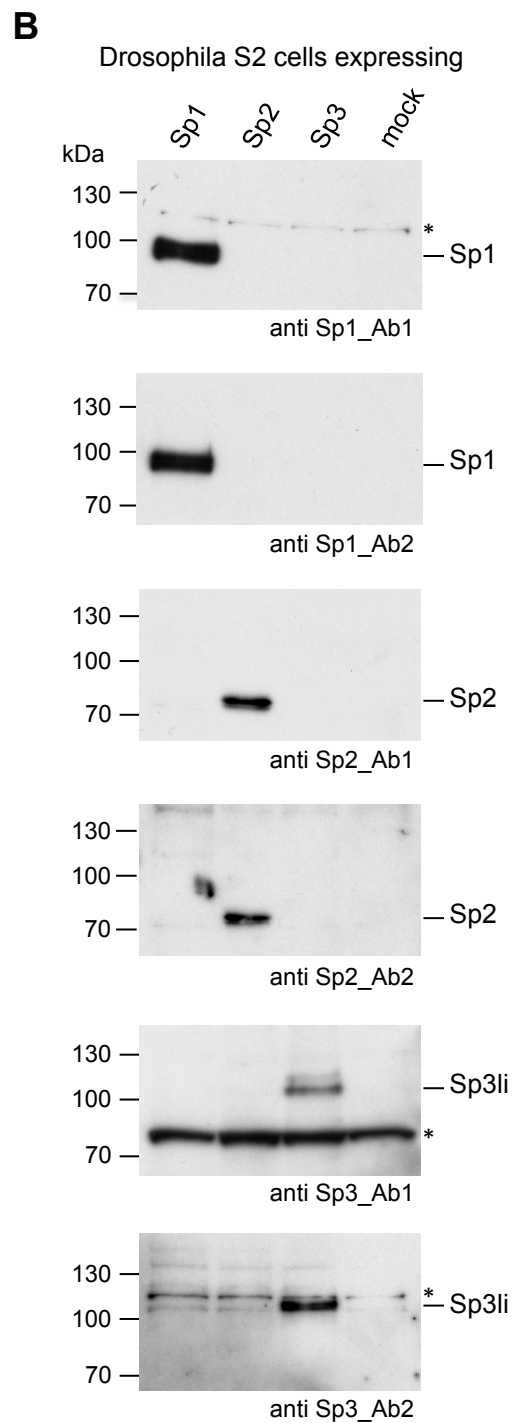

Supplement: S1 Fig — (A) Nuclear extracts of wt, Sp2ko and Sp3ko MEFs were probed with two different affinity-purified Sp1-, Sp2- and Sp3-specific antibodies. (B) Sp1, Sp2 and Sp3 were expressed in insect S2 cells lacking Sp factors. Subsequently, nuclear extracts were subjected to Western blot analysis using the affinity-purified Sp1-, Sp2- and Sp3-specific antibodies. The asterisks indicate non-specific bands. (PDF) [file pgen.1005102.s001.pdf]

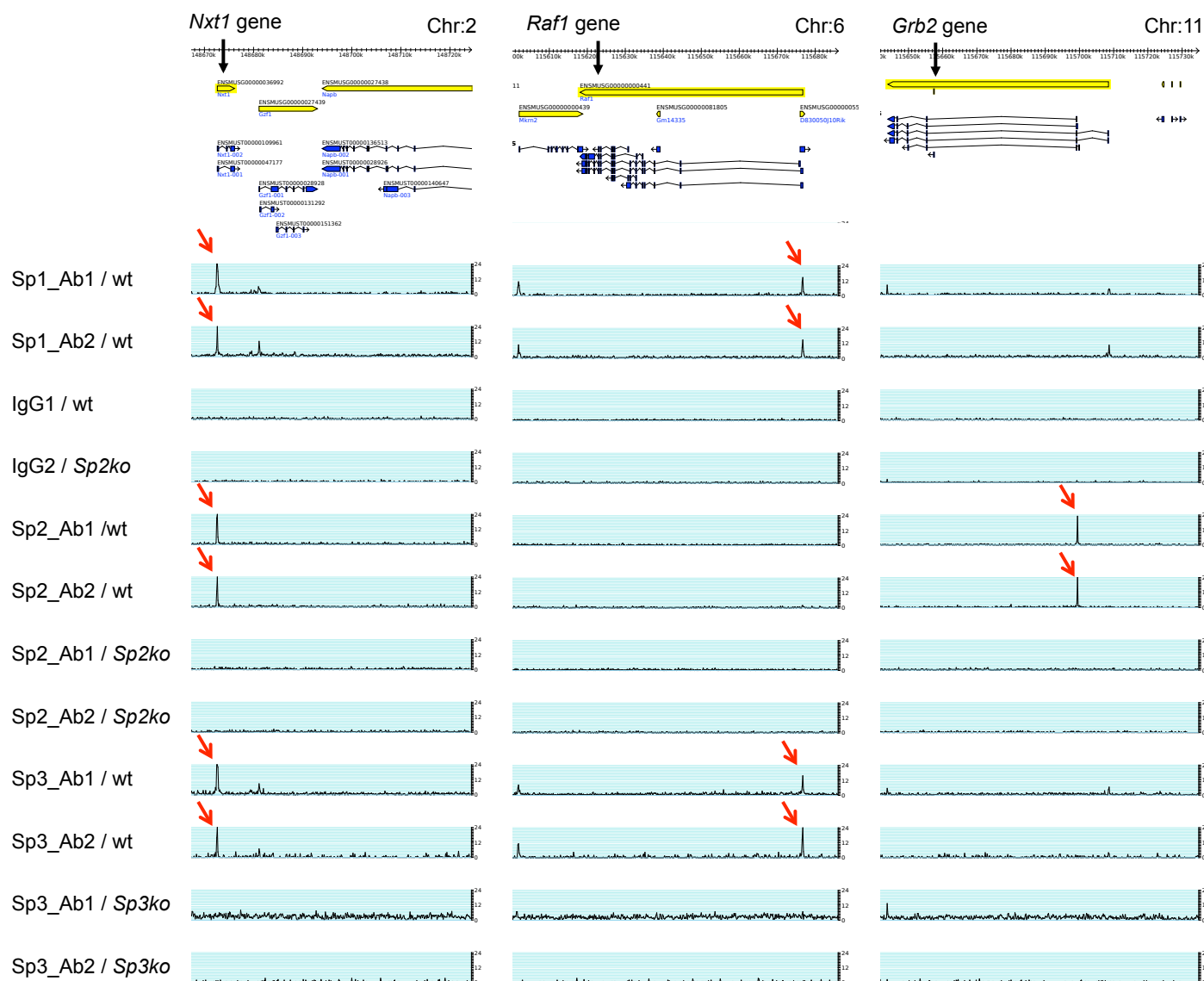

Supplement: S2 Fig — Shown are genomic binding patterns of Sp1, Sp2 and Sp3 at the Nxt1, Raf1 and Grb2 gene regions in wt, Sp2ko and Sp3ko MEFs. All three Sp factors are bound at the Nxt1 promoter, Sp1 and Sp3 but not Sp2 are bound at the Raf1 promoter, and Sp2 but not Sp1 and Sp3 is bound at the Grb2 downstream promoter. Due to the lack of Sp1ko MEFs, which are not viable, IgG ChIPs served as controls for the Sp1 ChIPs. (PDF) [file pgen.1005102.s002.pdf]

**A**

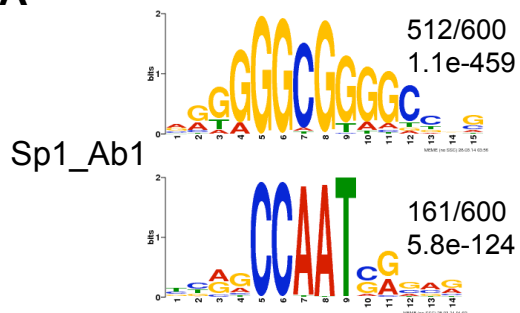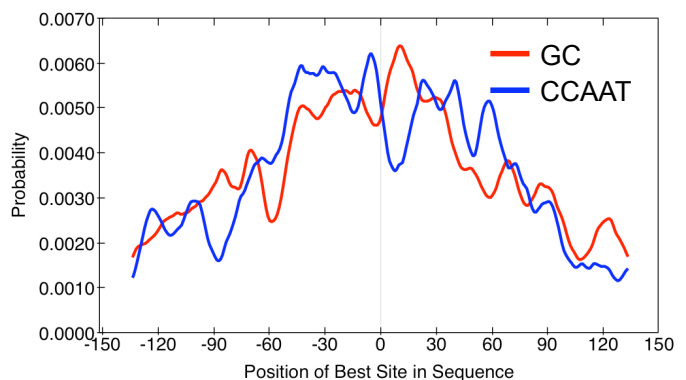

**B**

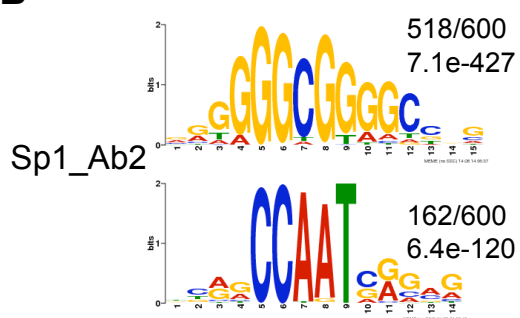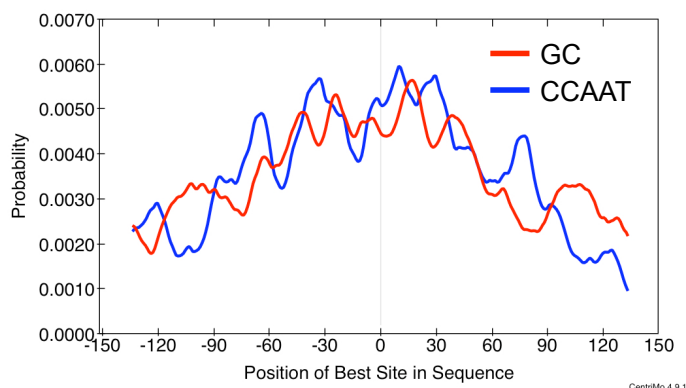

**C**

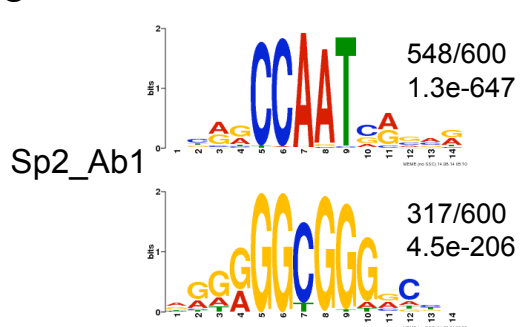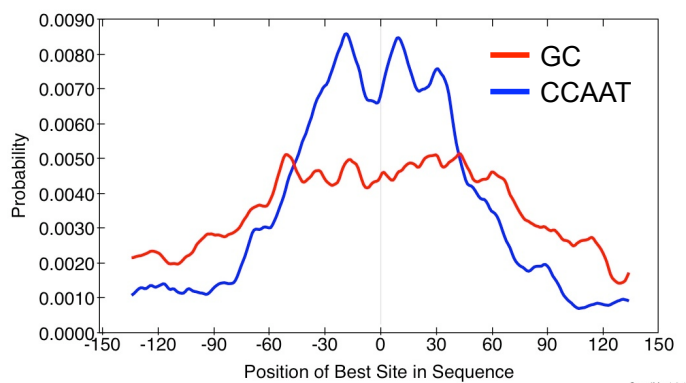

**D**

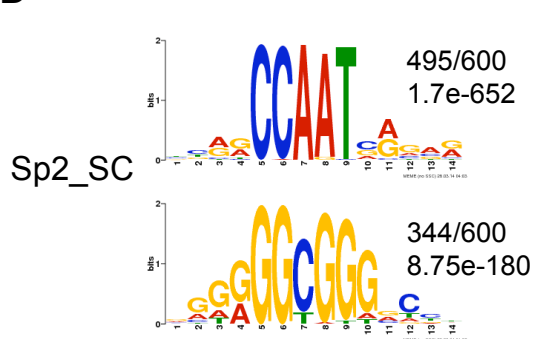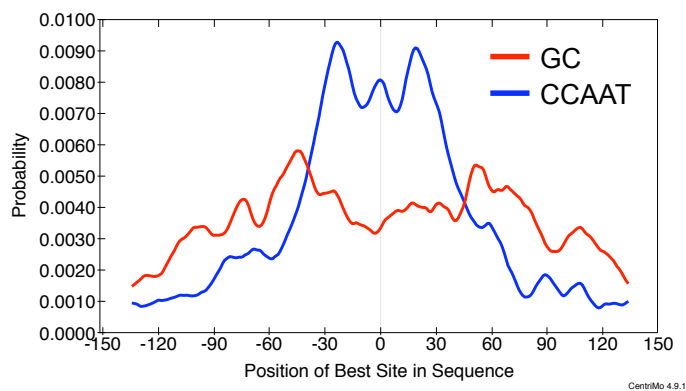

Supplement: S3 Fig — Sequence motifs (left) and their distribution (right) at Sp1 and Sp2 peaks in HEK293 cells were obtained by running MEME-ChIP [48] with 300 bp summits of the top 1000 Sp1 and Sp2 ChIP-seq peaks. (A) ChIP-seq with homemade Sp1 antibody 1. (B) ChIP-seq with homemade Sp1 antibody 2. (C) ChIP-seq with homemade Sp2 antibody 1 as published in [18]. (D) ChIP-seq with a commercial Sp2 antibody (Santa Cruz, sc-643). The numbers next to the logos indicate the occurrence of the motif (number of sites contributing to the construction of the motif) and the statistical significance (E-value). (PDF) [file pgen.1005102.s003.pdf]

**A**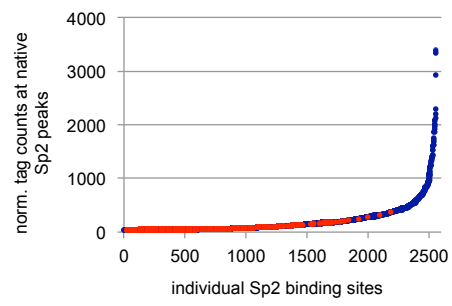**B**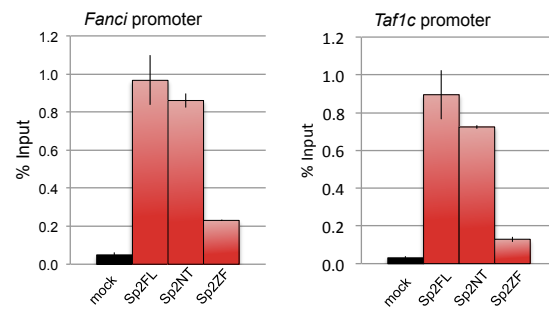

Supplement: S4 Fig — (A) Sites of Sp2 that are not co-bound by Nf-y represent low tag count Sp2 binding sites. Individual native Sp2 peaks in wt MEFs [18] were plotted against their normalized tag counts. Those sites that are not co-bound by Nf-y (see Fig. 7B) were overlaid with red dots. (B) Binding of Flag-tagged Sp2FL, Sp2ZF and Sp2NT to the Fanci and Taf1c promoters was analyzed by ChIP-qPCR. Anti-Flag antibodies were used for ChIP. The percent of input values are mean +/- SD (n = 3). (PDF) [file pgen.1005102.s004.pdf]
