# Supplementary material for: Zinc Finger Independent Genome-Wide Binding of Sp2 Potentiates Recruitment of Histone-Fold Protein Nf-y Distinguishing It from Sp1 and Sp3
Source: PLoS Genet. 2015 Mar 20;11(3):e1005102. doi: 10.1371/journal.pgen.1005102 (PMC4368557; doi:10.1371/journal.pgen.1005102)
Supplement: S5 Fig — Shown are genome browser snapshots. (PDF) [file pgen.1005102.s005.pdf]

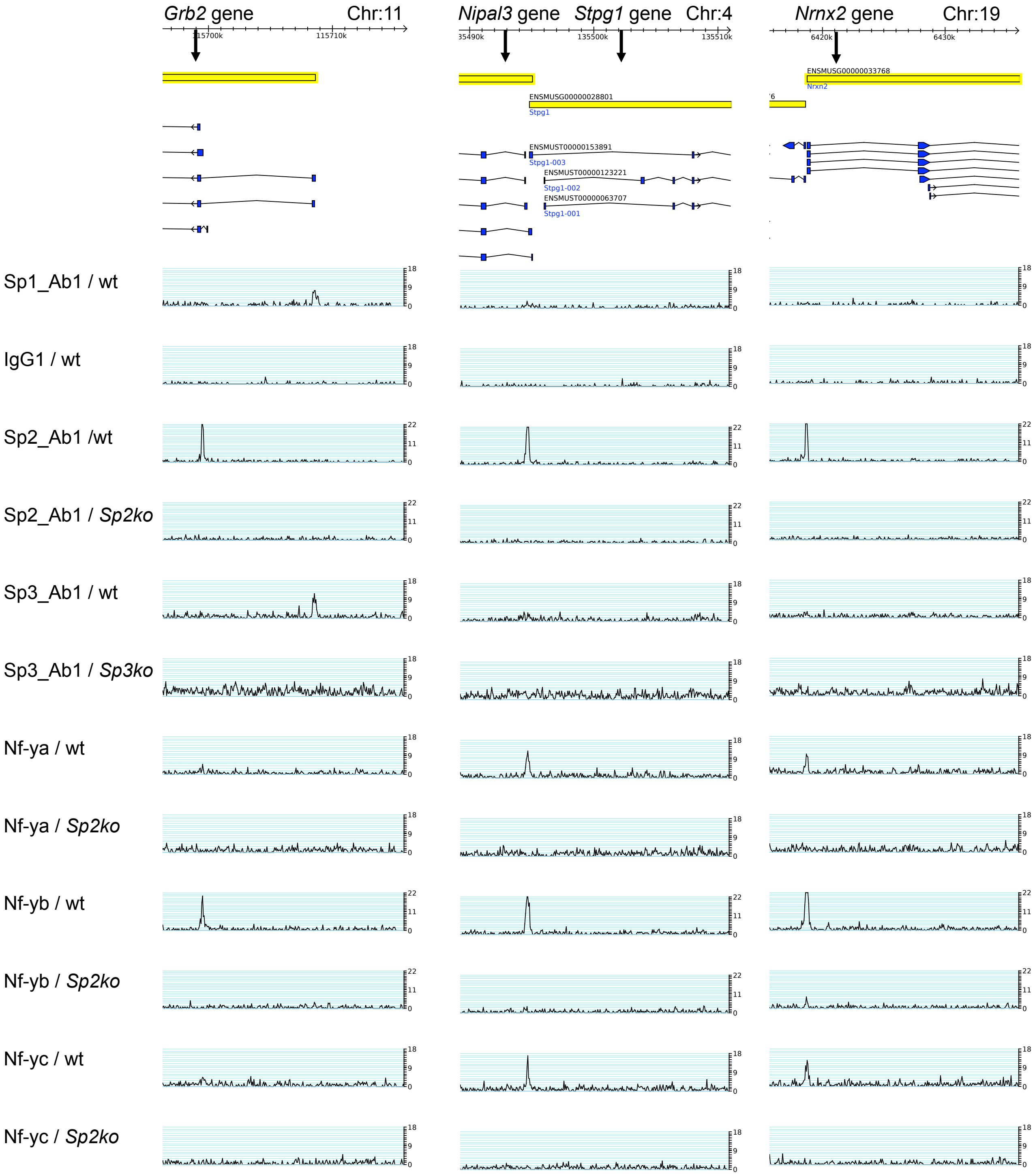

Grb2 gene
Chr:11
115700k
115710k
Nipal3 gene
Stpg1 gene
Chr:4
135500k
135510k
ENSMUSG00000028801
Stpg1
ENSMUST00000153891
Stpg1-003
ENSMUST00000123221
Stpg1-002
ENSMUST00000063707
Stpg1-001
Nrnx2 gene
Chr:19
6420k
6430k
ENSMUSG00000033768
Nrxn2
Sp1_Ab1 / wt
IgG1 / wt
Sp2_Ab1 /wt
Sp2_Ab1 / Sp2ko
Sp3_Ab1 / wt
Sp3_Ab1 / Sp3ko
Nf-ya / wt
Nf-ya / Sp2ko
Nf-yb / wt
Nf-yb / Sp2ko
Nf-yc / wt
Nf-yc / Sp2ko
