# Supplementary material for: Zinc Finger Independent Genome-Wide Binding of Sp2 Potentiates Recruitment of Histone-Fold Protein Nf-y Distinguishing It from Sp1 and Sp3
Source: PLoS Genet. 2015 Mar 20;11(3):e1005102. doi: 10.1371/journal.pgen.1005102 (PMC4368557; doi:10.1371/journal.pgen.1005102)
Supplement: S6 Fig — Shown are genome browser snapshots. (PDF) [file pgen.1005102.s006.pdf]

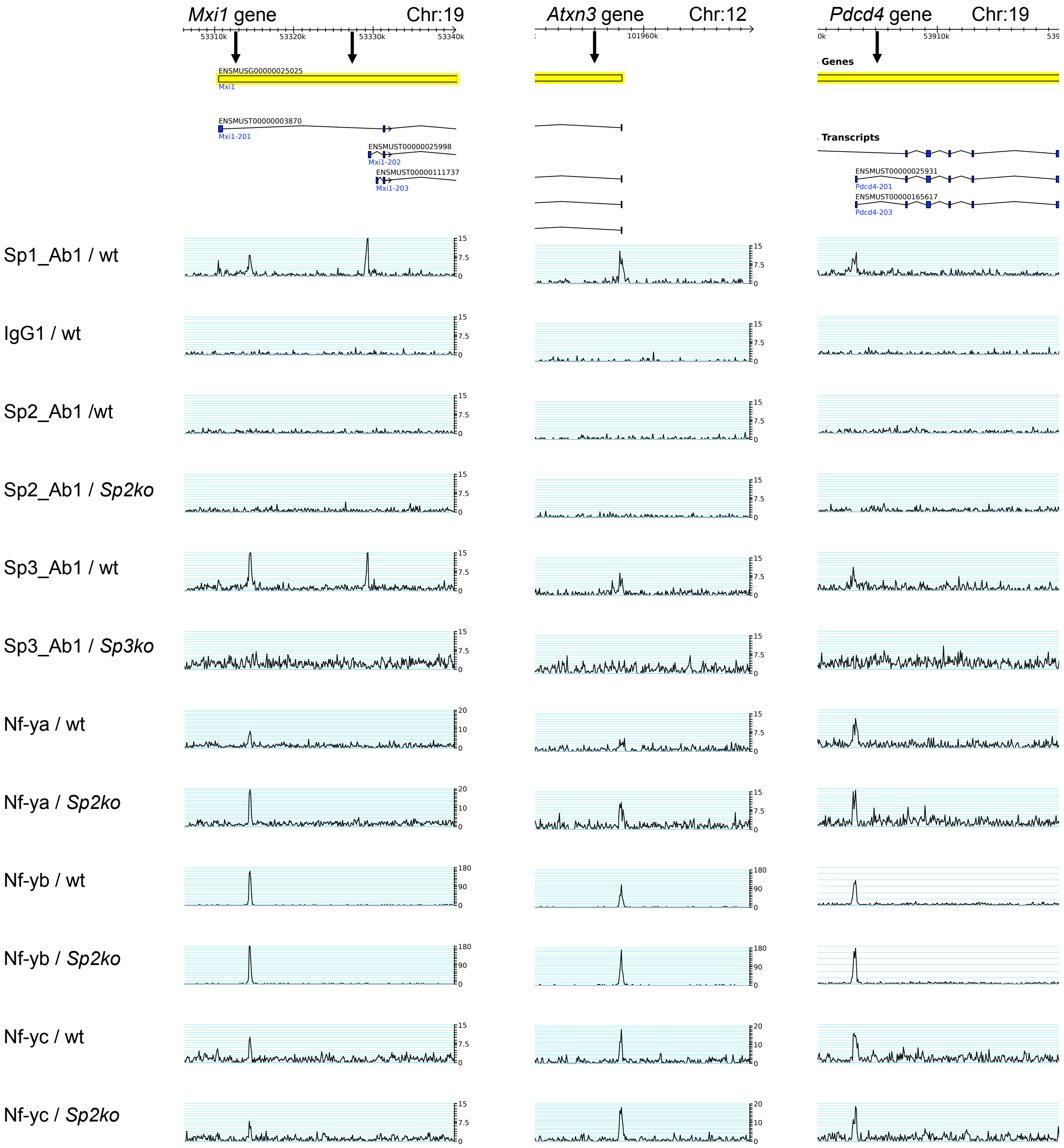
Mxi1 gene
Chr:19
53310k
53320k
53330k
53340k
ENSMUSG00000025025
Mxi1
ENSMUST00000003870
Mxi1-201
ENSMUST00000025998
Mxi1-202
ENSMUST00000111737
Mxi1-203
Atxn3 gene
Chr:12
101960k
Pdcd4 gene
Chr:19
53910k
Genes
Transcripts
ENSMUST00000025931
Pdcd4-201
ENSMUST00000165617
Pdcd4-203
Sp1_Ab1 / wt
IgG1 / wt
Sp2_Ab1 /wt
Sp2_Ab1 / Sp2ko
Sp3_Ab1 / wt
Sp3_Ab1 / Sp3ko
Nf-ya / wt
Nf-ya / Sp2ko
Nf-yb / wt
Nf-yb / Sp2ko
Nf-yc / wt
Nf-yc / Sp2ko
15
7.5
0
20
10
180
90
