# Supplementary material for: Zinc Finger Independent Genome-Wide Binding of Sp2 Potentiates Recruitment of Histone-Fold Protein Nf-y Distinguishing It from Sp1 and Sp3
Source: PLoS Genet. 2015 Mar 20;11(3):e1005102. doi: 10.1371/journal.pgen.1005102 (PMC4368557; doi:10.1371/journal.pgen.1005102)
Supplement: S7 Fig — The Sp box (yellow), the Btd box (blue) and the zinc finger region (bold) are highlighted. Colored letters within the N-terminal part highlight basic amino acids (K and R, violet), acidic amino acids (D and E, green) and glutamine residues (gray). (PDF) [file pgen.1005102.s007.pdf]

>Sp1

MSDQDHSMDEMTAVVKIEKGVGGNNGGNGNGGGAFSQARSSSTGSSSSTG
GGGQESQPSPLALLAATCSRIESPNENSNNSQGPSQSGGTGELDLTATQL
SQGANGWQIISSSSGATPTSKEQSGSSTNGSNGSESSKNRTVSGGQYVVA
AAPNLQNQQVLTGLPGVMPNIQYQVIPQFQTVDGQQLQFAATGAQVQQDG
SGQIQIIPGANQQIITNRGSGGNIIAAMPNLLQQAVPLQGLANNVLSGQT
QYVTNVPVALNGNITLLPVNSVSAATLTPSSQAVTISSSGSQESGSQPVT
SGTTISSASLVSSQASSSSFFTNANSYSTTTTTSNMGIMNFTTSGSSGTN
SQGQTPQRVSGLQGSDALNIQQNQTSGGSLQAGQQKEGEQNQQTQQQQIL
IQPQLVQGGQALQALQAAPLSGQTFTTQAISQETLQNLQLQAVPNSGPII
IRTPTVGPNGQVSWQTLQLQNLQVQNPQAQTITLAPMQGVSLGQTSSSNT
TLTPIASAASIPAGTVTVNAAQLSSMPGLQTINLSALGTSGIQVHPIQGL
PLAIANAPGDHGAQLGLHGAGGDGIHDDTAGGEEGENSPDAQPQAGRRTR
REACTCPYCKDSEGRGSGDPGKKKQHI**CHIQGCGKVYGKTSHLRAHLRWH**
TGERPFMCTWSYCGKRFTRSDELQRHKRTHTGEKKFACPECPKRFMRSDH
LSKHIKTHQNKKGGPGVALSVGTLPLDSGAGSEGSGTATPSALITTNMVA
MEAICPEGIARLANSGINVMQVADLQSINISGNGF

pKi=4.4

>Sp2

MSDPQTSMAATAAVSPSDYLQPAASTTQDSQPSPLALLAATCSKIGPPAV
EAAVTPPAPPQPTPRKLVPIKPAPLPLSPGKNSFGILSSKGNILQIQGSQ
LSASYPGGQLVFAIQNPTMINKGTRSNANIQYQAVPQIQASNSQTIQVQP
NLTNQIQIIPGTNQAIITPSPSSHKPVPIKPAPIQKSSTTTTPVQSGANV
VKLTGGGGNVTLTLPVNNLVNASDTGAPTQLLTESPPTPLSKTNKKARKK
SLPASQPPVAVAEQVETVLIETTADNIIQAGNNLLIVQSPGGGQPAVVQQ
VQVVPPKAEQQQVVQIPQQALRVVQAASATLPTVPQKPSQNFQIQAAEPT
PTQVYIRTPSGEVQTVLVQDSPPATAAATSNTTCSSPASRAPHLSGTSKK
HSAAILRKERPLPKIAPAGSIISLNAAQLAAAAQAMQTININGVQVQGVP
VTITNTGGQQQLTVQNVSGNNLTISGLSPTQIQLQMEQALAGETQPGEKR
RRMACTCPNCKDGEKRSGEQGKKKHV**CHIPDCGKTFRKTSLLRAHVRLHT**
GERPFVCNWFFCGKRFTRSDELQRHARTHTGDKRFECAQCQKRFMRSDHL
TKHYKTHLVTKNL

pKi=10.6

>Sp3

MTAPEKPVKQEEMAALDVDSGGGGGGGGHGEYLQQQQQHGNGAVAAAAA
AQDTQPSPLALLAATCSKIGPPSPGDDEEEAAAAAGAPAAAGATGDLASA
QLGGAPNRWEVLSATPTTIKDEAGNLVQIPSAATSSGQYVLPLQNLQNQQ
IFSVAPGSDSSNGAVSSVQYQVIPQIQSADGQQVQIGFTGSSDNGGINQE
SSQIQIIPGSNQTLLASGTPSANIQNLIPQTGQVQVQGVAIGGSSFPGQT
QVVANVPLGLPGNITFVPINSVDLDSLGLSGSSQTMTAGINADGHLINTG
QAMDSSDNSERTGERVSPDINETNTDTDLFVPTSSSSQLPVTIDSTGILQ
QNTNSLTTSSGQVHSSDLQGNYIQSPVSEETQAQNIQVSTAQPVVQHLQL
QESQQPTSQAQIVQGITPQTIHGVQASGQNISQQALQNLQLQLNPGTFLI
QAQTVTPSGQVTWQTFQVQGVQNLQNLQIQNTAAQQITLTPVQTLTLGQV
AAGGAFTSTPVSLSTGQLPNLQTVTVNSIDSAGIQLHPGENADSPADIRI
KEEEPDPEEWQLSGDSTLNTNDLTHLRVQVVDEEGDQQHQEGKRLRRVAC
TCPNCKEGGGRGTNLGKKKQHI**CHIPGCGKVYGKTSHLRAHLRWHSGERP**
FVCNWMYCGKRFTRSDELQRHRRTHTGEKKFVCPECSKRFMRSDHLAKHI
KTHQNKKGIHSSSTVLASVEAARDDTLITAGGTTLILAKIQQGSVSGIGT
VNTSATSNQDILTNTEIPLQLVTVSGNETME

pKi=3.9
